# Genome wide identification of wheat and Brachypodium type one protein phosphatases and functional characterization of durum wheat TdPP1a

**DOI:** 10.1371/journal.pone.0191272

**Published:** 2018-01-16

**Authors:** Mariem Bradai, Habib Mahjoubi, Andrea Chini, Marie-Edith Chabouté, Moez Hanin, Chantal Ebel

**Affiliations:** 1 Laboratory of Biotechnology and Plant Improvement, Center of Biotechnology of Sfax, Sfax, Tunisia; 2 Institut de Biologie Moléculaire des Plantes, CNRS, Université de Strasbourg, Strasbourg, France; 3 Plant Molecular Genetics Department, Centro Nacional de Biotecnologia-CSIC (CNB-CSIC), Madrid, Spain; 4 Plant Physiology and Functional Genomics Research Unit, Institute of Biotechnology, University of Sfax, Sfax, Tunisia; National Institute of Plant Genome Research, INDIA

## Abstract

Reversible phosphorylation is an essential mechanism regulating signal transduction during development and environmental stress responses. An important number of dephosphorylation events in the cell are catalyzed by type one protein phosphatases (PP1), which catalytic activity is driven by the binding of regulatory proteins that control their substrate specificity or subcellular localization. Plants harbor several PP1 isoforms accounting for large functional redundancies. While animal PP1s were reported to play relevant roles in controlling multiple cellular processes, plant orthologs remain poorly studied. To decipher the role of plant PP1s, we compared *PP1* genes from three monocot species, Brachypodium, common wheat and rice at the genomic and transcriptomic levels. To gain more insight into the wheat PP1 proteins, we identified and characterized *TdPP1*a, the first wheat type one protein phosphatase from a Tunisian durum wheat variety Oum Rabiaa3. TdPP1a is highly conserved in sequence and structure when compared to mammalian, yeast and other plant PP1s. We demonstrate that TdPP1a is an active, metallo-dependent phosphatase *in vitro* and is able to interact with AtI2, a typical regulator of PP1 functions. Also, TdPP1a is capable to complement the heat stress sensitivity of the yeast mutant indicating that TdPP1a is functional also *in vivo*. Moreover, transient expression of TdPP1a::GFP in tobacco leaves revealed that it is ubiquitously distributed within the cell, with a strong accumulation in the nucleus. Finally, transcriptional analyses showed similar expression levels in roots and leaves of durum wheat seedlings. Interestingly, the expression in leaves is significantly induced following salinity stress, suggesting a potential role of TdPP1a in wheat salt stress response.

## Introduction

Reversible phosphorylation is one of the most common post-translational modifications described in eukaryotes. The plant cell has therefore evolved plethora of kinases and phosphatases that participate in regulating accordingly and specifically multiple signaling processes. Depending on the residues they dephosphorylate, one can distinguish Ser/Thr phosphatases (PSTPs) from Tyrosine phosphatases (PTPs) and Double Specificity Phosphatases (DSPs) which are able to dephosphorylate Ser, Thr and Tyr [1–4]

Type one protein phosphatases (PP1s) are Ser/Thr phosphatases highly conserved in sequence and structure among eukaryotes with more than 70% of the residues of the core catalytic domain being identical between animals and plants PP1s [2]. Beyond this strong conservation, PP1s are characterized by their functional diversity [5, 6] as they participate in multiple biological processes such as cell cycle regulation, glucose metabolism, transcription, protein synthesis and light signaling.

To ensure these multiple functions, the catalytic subunits of PP1s which are solely inactive, interact with regulatory proteins to determine substrate specificity or subcellular localization. More than 200 interacting proteins have been described in mammals and most of them harbor a conserved motif (called RVxF for [R/K][R/K][V/I]x[F/W]) that binds to a well described hydrophobic groove of PP1s [7, 8]. Most of PP1-interacting proteins (PIPs) are also described as intrinsically unstructured proteins (IUPs) [9].

Mammalian cells have three PP1 isoforms: PP1α, PP1β (also called PP1δ) and PP1γ [10, 11]. *Arabidopsis* contains nine isoforms called TOPPs (Type One protein Phosphatase), while rice and *Vicia Faba* have five and four different PP1s respectively [12, 13]. Interestingly, *Saccharomyces cerevisiae* has a single essential gene *GLC7* [14, 15] which functions in cellular integrity, morphology, glucose metabolism, meiosis and kinetochore/microtubule binding but also endocytosis [14, 16].

Knowledge about functional contribution of plant PP1 has been increasing only lately. In 2006, [13] studied expression and function of *Vicia faba* VfPP1c-1, -2, -3 and -4 and showed that *VfPP1c-1* expression is restricted to guard cells, leaves and roots but excluded from stems and mesophyl cells. In stomata, VfPP1c-1 is responsible for their opening upon blue light signaling acting downstream of phot1 and phot2 [13]. The analysis of the *Arabidopsis* dominant-negative mutant *topp4-1* revealed the role of TOPP4 in gibberellic acid (GA) pathway [17]. TOPP4 by dephosphorylating DELLAs activates their degradation by the 26S proteasome launching GA responses [17]. Moreover, TOPP4 stops degradation of PIF5 (PHYTOCHROME-INTERACTING FACTOR5) participating in light signaling [18] and regulates auxin gradient at the epidermis by dephosphorylating PIN1 [19]. *Arabidospis* TOPP1 is involved in ABA signaling by inhibiting the activity of SnRK2 kinase together with AtI2 [20]. AtI2, considered as a negative regulator of TOPP activities [21], acts here to promote TOPP1-SnRK2 binding [20].

In rice, RSS1 (RICE SALT SENSITIVE 1) protein was reported to interact with OsPP1a, most likely to control meristem maintenance upon salt stress [12, 22] and overexpression of OsPP1a confers salinity tolerance to rice plants [23]. Deciphering the pleiotropic roles of PP1 in plants is difficult because of the high diversity of PIPs driving a wide range of substrates and because of the increased number of PP1 isoforms that are redundant in subcellular location and expression pattern.

The present study deals with the identification of Brachypodium and common wheat PP1 genes, comparing their genomic organization, their protein homologies and their expression in different tissues and under different conditions. In addition, we report the isolation and characterization of the first durum wheat type one protein phosphatase called TdPP1a. Our data reveal that TdPP1a shares the common primary sequence characteristics with yeast, mammalian, and plant PP1s. More interestingly, TdPP1a has a metallo-dependent phosphatase activity *in vitro* and is able to complement yeast orthologous mutant. Furthermore, the transcriptional induction of TdPP1a by NaCl treatments suggests that it may play a regulatory role in wheat salt stress response.

## Materials and methods

### Plant material and stress treatments

Seeds of Tunisian durum wheat variety Oum Rabiaa3 provided from INRAT (Tunisian Agronomic Research Institute) were surface sterilized with 1.5% (v/v) sodium hypochlorite for 15 min with gentle agitation, rinsed three times with sterile water and grown on wet Whatman paper, for 2 days in the dark, and for a week in a growth chamber at 23°C, under a 16 h photoperiod (16 h day/8 h night) and with 60% relative humidity. Plants were watered with distilled water three times a week. Stress treatments were done on 10-day-old seedlings using 150 mM NaCl, 15% polyethylene glycol 8000 (PEG 8000) for 24 h. Cold stress was performed by putting the seedlings at 4°C for 24h.

### Identification of Brachypodium distachyon and Triticum aestivum PP1 families

Genomic, cDNA and protein sequences were retrieved by combining HMMER and Psi-BLAST searches [24] using *Oryza sativa* PP1s as queries on TGACv1 genome (The Genome Analysis Center Version 1) from EnsemblPlant (http://plants.ensembl.org/Triticum_aestivum/Info/Index) and keyword searches within phytozome database (https://phytozome.jgi.doe.gov). Retrieved sequences were aligned with rice PP1s using MEME (http://meme-suite.org/index.html) to search for conserved motifs. FIMO was further used using the N-terminal S/T protein phosphatase motif as query to search for additional PP1 proteins (Pfam PF16981.4/Interpro IPR 031675; [4]). Ultimate searches were done on EnsemblPlant to find orthologs and paralogs of wheat PP1s in *Brachypodium distachyon*, *Oryza sativa*, *Triticum urartu* and *Aegilops tauschii*. cDNA sequences of wheat and Brachypodium identified herein are available in S1 Fig. Genomic and cDNA sequences of Brachypodium, wheat and rice have been used to define PP1 gene structure using GSDS tool (http://gsds.cbi.pku.edu.cn/index.php). Arabidopsis type one protein phosphatases were retrieved using TAIR and phytozome databases.

Protein parameters (Molecular weight, pI, Instability Index, GRAVY) have been computed by protparam (https://web.expasy.org/protparam/) and are presented in S2 Table.

### Phylogenetic analyses

Multiple alignment and phylogenetic analyses were conducted using MEGA6.06 software [25]. Multiple alignments of proteins were done using ClustalW and the BLOSUM matrix with default settings. The phylogenetic tree was created using the Neighbor-joining method based on the number of aa substitutions per site and the bootstrap test with 1000 replicates. Ka/Ks calculation was done using MEGA 6.06 and the Nei-Gojobori method with p-values>0.05.

### Expression analyses

RNA Seq data were downloaded from WheatExpress, BAR and Phytomine. Differential expression was analyzed using BAR HeatMapper (http://bar.utoronto.ca/ntools/cgi-bin/ntools_heatmapper.cgi) with log2 transformed FPKM values.

### Statistical analyses

The statistical analyses were performed using Excel Student’s t-test. *p* values <0.01 were considered as statistically significant.

### TdPP1a isolation and cloning

Using cDNA sequences of *Oryza sativ*a OsPP1a and EST sequences of *Triticum aestivum* available in databases (The Gene Index: http://compbio.dfci.harvard.edu/tgi/) primers were designed for PCR amplification. A first PCR amplification using TdPP1F2 (5’-ACTCCACCGATCTCTCTCCA-3) and TdPP1R2 (5’-ACTAACCTCAGAGCCTCTAG-3) was done on wheat cDNA of Oum Rabiaa3 Tunisian durum wheat variety by adding 1% DMSO using the following program: 94°C, 30 s; 50°C, 30 s; 72°C 1 min in a final volume of 20 μl. One microlitre of this first PCR product was used for nested PCR amplification using TdPP1F1 (5’-TGCAGCTCTCCGAGTCGGAG-3) and TdPP1R1 (5’-TCAGCAGGTTTGAGAATTTG-3’) 1% DMS0 and an annealing temperature of 52°C. A single band was observed of approximately 1000 bp that was cloned into the pCR2.1 vector (Invitrogen) giving rise to the pPP1FL2 clone. After sequencing using the dye terminator cycle sequencing method (Applied Biosystems), BLAST analyses were performed [24]. The PP1 sequence is deposited in GenBank under accession KM203893.

### RNA isolation and TdPP1 expression analyses

Total RNA was prepared from wheat leaves and roots tissues using TRIzol (Invitrogen) as recommended by the manufacturer. RNA was then treated with DNaseI (Thermo Scientific) at 37°C for 15 min. DNase-treated RNA samples (1 μg) were reverse-transcribed using M-MLV reverse transcriptase (Invitrogen). The reverse transcription (RT) reactions were performed at 37° C for 1 h using 2 μM oligodT. The resulting RT-reaction products were then used as template for PCR amplification generating a TdPP1 specific fragment of 200 bp using the following primers: TdPP1F5 5’-CTGTGGCCGCTCTAATTGAT-3’ and TdPP1R5 5’-TGTGAATGAAACGGCCCTAT-3’. A wheat Actin gene fragment used as an internal control was amplified with the primers ActF: 5’-GGCGATGAAGCTCAATCCAAACG-3’ and ActR: 5’-GGTCACGACCAGCAAGATCAAGACG-3’. The PCR products were separated by electrophoresis in 1% agarose gel.

Quantitative RT-PCR was performed on RNA from aerial parts of 7-day old wheat seedlings treated either with NaCl 150 mM or ABA 100 μM for an hour. The RNA was cleaned up from DNA contamination using on-column DNAse I removal kit (Roche). 1 μg of total RNA was used for reverse transcription using cDNA synthesis kit (Roche). After 1/10^th^ dilution, 5 μl of cDNA was used as a template for QPCR analyses in a total volume of 15 μl using Power SYBR Master mix (Applied Biosystems). Amplification and quantification was performed in a 7500 Real Time PCR system (Applied Biosystems) and using the above-mentioned primer pairs for TdPP1a and actin. Wheat Actin was used as internal control. Quantification was performed using the ΔΔCt method [26] using actin and time 0 as references.

### Expression and purification of the recombinant TdPP1 protein in *Escherichia coli*

TdPP1 open reading frame (ORF) was cloned in-frame with a 6xHis tag into the pET28a expression vector (pHis::TdPP1). For expression, *E*. *coli* BL21 (DE3) cells were transformed with pHis::TdPP1 and grown overnight in LB medium containing 50 μg/ml kanamycin. Overnight culture was diluted to an OD_600nm_ = 0.1 on the following morning in 300 ml of LB supplemented with kanamycin, and grown until OD_600nm_ reaches 0.6–0.8. The production of the recombinant protein was induced by adding 1 mM IPTG (isopropyl β-D-thiogalactopyranosid) overnight at 30°C. The next day, cells were harvested by centrifugation (4000 rpm for 15 min at 4° C) and resuspended in 150 ml lysis buffer (20 mM Tris-HCl (pH 8), 100 mM NaCl, 5 mM imidazole, 1 mM PMSF, 0.5% NP-40). The cells were then subsequently lysed by sonication on ice. After a centrifugation at 10000 rpm for 20 min at 4° C, recombinant protein was purified by affinity chromatography on nickel columns (His60 Ni Superflow Resin Clontech). After four washes with 20 mM Tris-HCl (pH 8), 100 mM NaCl, with increasing imidazole (10 to 40 mM) concentrations, the tagged protein was eluted with 20 mM Tris-HCl (pH 8), 100 mM NaCl, 0.5 M imidazole. All these steps were performed at 4°C.

### Phosphatase activity

The phosphatase assay was performed according to [27] using 1 μg of purified His::TdPP1 in the presence or absence of Mn^2+^ and Fe^2+^ using the 3-O-methylfluorescein phosphate (OMFP, Sigma Aldrich) as substrate. Phosphatase activity was measured at 30° C after 30 min in 0.8 ml of reaction buffer (50 mM Tris-HCl (pH 8), 150 mM NaCl, 500 μM OMFP). The amount of 3-O-methylfluorescein (OMF) product was measured by absorbance at 477 nm.

### Subcellular localization of TdPP1 protein

To investigate the subcellular localization of TdPP1 protein, a gateway cloning was carried out to have a C-terminal protein fusion with GFP. pENTR-TdPP1 construct was recombined into the destination vector, pB7FWG2 using LR clonase (Invitrogen) as recommended by the manufacturer. After spectinomycin selection, the recombinant pB7FWG2-TdPP1 clone verified by PCR and sequencing. This construct was mobilized in *Agrobacterium tumefaciens* strain GV3101 by the freeze-thaw method [28]. *Agrobacterium*-mediated transient expression in tobacco leaf epidermal cells was performed as follows: *A*. *tumefaciens* containing pB7FWG2-TdPP1 were grown overnight in LB medium supplemented with 50 μg/ml rifampicin and 100 μg/ml spectinomycin. The cells were collected by centrifugation for 15 min at 2500 rpm and resuspended in infiltration buffer (10 mM MES pH 5.5, 10 mM MgSO_4_, and 200 μM acetosyringone) to final OD_600_ = 0.6 and incubated at 25°C in the dark for 2 h before agroinfiltration of tobacco leaves using 1 ml syringes. About 72 h after agroinfiltration, 1 cm^2^ leaf explants were prepared, and GFP fluorescence was observed in intact epidermal cells with 40x and 63x objectives using a confocal laser scanning microscope (Zeiss).

### Transformation of yeast strains and growth assays

The pPP1FL2 construct was digested with *Xba*I releasing the TdPP1 ORF then inserted into the *Xba*I site of the yeast expression vector pYES2 vector. The resulting plasmid pYES2-TdPP1 and the empty pYES2 were then introduced into *Saccharomyces cerevisiae* cells of using the PEG/LiAC transformation method as described [29]. The yeast strains used in this assay were the following: KT1112 (*MAT a his3 leu2 ura3-52*; [30]) and KT 2210/*glc7F256A* (*MAT a his3 leu2 trp1 ura3-52 glc7-F256A*; [31]). The recombinant yeast clones were grown overnight in 2 ml YPD at 30°C. On the next day, OD_600nm_ of each culture was measured and diluted in rich medium containing galactose to an OD_600nm_ = 0.2. After 2h of growth at 30°C, ten times dilution series (10^−1^ to 10^−4^) were used to evaluate sensitivity of transformants to heat stress treatments (37°C) by growth on plates containing YPGalactose (drop tests) as previously described [32]. For stress treatments in liquid media, the recombinant yeast clones were grown overnight in 2 ml YPD at 30°C. On the next day, the cultures were diluted in YPGalactose to an OD_600nm_ = 0.1, and grown at 30°C or 37°C. Growth of each sample was monitored by OD_600nm_ measurements after 24 h.

### Yeast-two-hybrid interactions

*At*I2 cloned in pDON221 was kindly given by Dr. Greg Moorhead (University of Calgary, Canada). This clone was used to clone *At*I2 in frame with the GAL4 Activation Domain of pGAD vector using the LR Recombinase (Invitrogen) and the manufacturer’s protocol. In parallel, LR recombination of pENTR::PP1 with pGBKT7 vector allowed the cloning of PP1 in frame with the GAL4 DNA binding-domain.

Yeast strain PJ69-4A was co-transformed with the combination of constructs as described [29]. Co-transformants were selected on SD-medium lacking Leu and Trp. Interactions were tested by spotting at least 4 co-transformants for each combination on SD-medium without Leu and Trp, without Leu, Trp, His, or without Leu, Trp, His and Ade supplemented or not by 3-Amino 1, 2, 4 Triazole (3-AT; 2 mM).

## Results

### Identification of Brachypodium and common wheat *PP1* genes

Within monocotyledonous species, 5 isoforms of type 1 protein phosphatases have been described in *Oryza sativa* [12] but for other monocots little information was available. We therefore characterized this family in key graminae species, wheat and Brachypodium. Using the 5 different rice isoforms (OsPP1s) as queries for HMMER analyses, we searched for bread wheat and Brachypodium homologous genes and proteins using the Ensembl Plant and phytozome databases (http://plants.ensembl.org/Triticum_aestivum/Info/Index; http://phytozome.jgi.doe.gov). For common wheat, 18 different protein-coding genes could be identified whereas 8 were identified for Brachypodium. The eight Brachypodium PP1 genes were located on chromosome 1 (n = 3), chromosome 2 (n = 3) and chromosome 3 (n = 2) and encoded 8 different proteins. Within the eighteen *T*. *aestivum* PP1 genes, 3 were located on chromosome 1, 5 on chromosome 3, 4 on chromosome 4, 2 on chromosome 5 and 4 on chromosome 6. These 18 different genes encoded 6 different PP1 isoforms.

**Fig 1:**
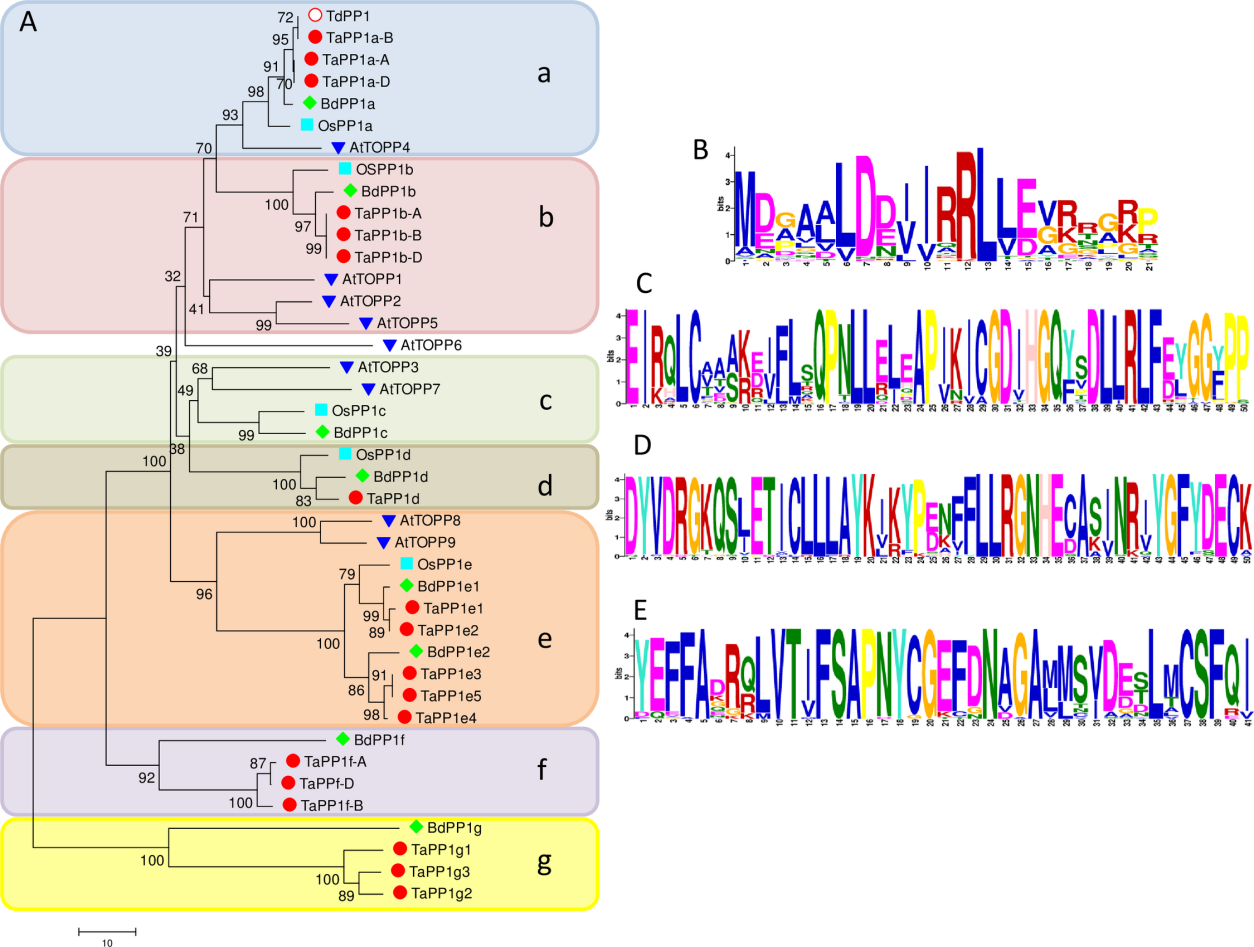
Phylogenetic analyses of wheat, Brachypodium, rice and Arabidopsis PP1 proteins. (A) Phylogenetic tree was obtained using MEGA6 with the Neighbor-Joining method based on protein sequences from EnsemblPlant, Uniprot, phytozome and TAIR. TdPP1a: *T*. *durum* PP1 (KM203893). Wheat, Brachypodium and rice identifiers are indicated in Table 1. Wheat proteins are indicated with red dots, rice proteins with light blue squares, Brachypodium PP1 with green diamonds and Arabidopsis with dark blue triangle. The percentage of replicate trees (bootstrap test with 1000 replicates) in which each taxa clustered together is indicated on each branch. The tree is drawn to scale based on evolutionary distances in number of amino acid substitutions per site. Sequence logos of N-terminal S/T phosphatase (B) and conserved motifs within the catalytic core (C, D, E) were obtained obtained using MEME on multiple alignments of rice, Brachypodium and wheat PP1 proteins.

Fig 1 shows a phylogenetic tree constructed using MEGA6 and the Neighbor-Joining method to study the evolutionary relationship between the PP1 proteins from rice, Brachypodium, *T*. *aestivum* and the dicot *Arabidopsis*. Remarkably, the different proteins are not clustered according to their species of origin. Indeed the five OsPP1 do not cluster together but belong to distinct clusters with the different proteins of wheat and Brachypodium. Arabidopsis TOPPs are more divergent as they form distinct clusters (Fig 1).

For both graminae, as shown in Fig 1, orthologous proteins of OsPP1a, OsPP1b, OsPP1d and OsPP1e could be found. In *B*. *distachyon* an OsPP1c orthologous protein has been identified but no ortholog of *T*. *aestivum* could be found perhaps because the genome is only partially sequenced. Interestingly, within the wheat and *Brachypodium* orthologs of OsPP1e we identified two distinct groups indicating that wheat and Brachypodium may have two different isozymes for this member (Fig 1). Furthermore, our analysis revealed that wheat and Brachypodium possess one or two more divergent PP1 isoforms clustered around BdPP1f/Bradi1g00690 and BdPP1g/Bradi2g03597 (Cluster f and g; Fig 1). TaPP1g is encoded by three genes located on chromosome 3B where *TaPP1g1* and *TaPP1g2* are separated by 80 kB on the same contig indicating possible gene duplications. *TaPP1g1*, *g2* and *g3* are indicated as paralogs by EnsemblPlant with identities ranging between 85.6 and 94.6%.

*TaPP1a*, *TaPP1b* and *TaPP1e* genes are located on the three genomes (AABBDD) (Fig 1). For these genes, genomic and cDNA alignments showed high identities (>95%) indicating redundancy over the different subgenomes. PBLAST searches on common wheat ancestors *Triticum urartu* (AA) and *Aegilops tauschii* (DD) allowed the identification of proteins with identities ranging between 89.8% and 100% (S1 Table).

Further analyses indicated that rice, Brachypodium and wheat proteins within each cluster were conserved in size (from 296 to 354 amino acids long; Table 1), their molecular weight were ranging from 32.4 to 37.9 kDa (S2 Table), each protein-groups differing in the size of the N-terminal domain. Most of rice, Brachypodium and wheat phosphatases have a calculated isoelectric point close to 5 (5.03–5.48) indicating that they are composed of more negatively charged amino acids than positively charged ones. Surprisingly, the f isozymes of Brachypodium and common wheat have a pI of ≈7 (*ie*. 7.05–7.08) with equal numbers of acidic and basic amino acids (S2 Table).

**Table 1:** Wheat, Brachypodium and rice type one protein phosphatases.

Gene Name	Gene ID	Protein size (aa)	Oryza sativa orthologs	Brachypodium orthologs
TaPP1a-A	Traes_4AS_6D7CDA716	321	OsPP1a Os03g0268000	Bradi1g66970 BdPP1a
TaPP1a-B	Traes_4BL_3AA55AD10	320		
TaPP1a-D	Traes_4DL_350C0974E	321		
TaPP1b-A	Traes_6AL_CCB16DE7E	320	OsPP1b Os02g0820000	Bradi3g55614 BdPP1b
TaPP1b-B	Traes_6BL_93357D848	364		
TaPP1b-D	Traes_6DL_82B22A082	324		
NF	NF		OsPP1c Os06g0164100	Bradi1g48410 BdPP1c
TaPP1d	Traes_6AL_DC03CC56C	304	OsPP1d Os08g0455600	Bradi3g37570 BdPP1d
TaPP1e1	Traes_3AS_8B6A13B23	318	OsPP1e Os01g0349400	Bradi2g12650 BdPP1e1
TaPP1e2	Traes_3B_9B97F74FC	352		
TaPP1e3	Traes_1AS_4025A300E	325		Bradi2g35150 BdPP1e2
TaPP1e4	Traes_1BS_BF71914E7	325		
TaPP1e5	Traes_1DS_309F807A1	335		
TaPP1f-A	Traes_4AL_2EBB63DBA	326	NF	Bradi1g00690 BdPP1f
TaPP1f-B	Traes_5BL_86F86B4A9	326	NF	
TaPP1f-D	TRIAE_CS42_5DL_TGACv1_433240_AA1406660.1	325	NF	
TaPP1g1	TRIAE_CS42_3B_TGACv1_221851_AA0751720.1	327	NF	Bradi2g03597 BdPP1g
TaPP1g2	TRIAE_CS42_3B_TGACv1_221851_AA0751750.1	326	NF	
TaPP1g3	TRIAE_CS42_3B_TGACv1_224143_AA0792930.1	296	NF	

Gene IDs starting with Traes are from phytozome https://phytozome.jgi.doe.gov/pz/portal.html, Gene IDs starting with TRIAE are from EnsemblPlant. NF: Not found

On another hand, multiple alignment of rice, Brachypodium and wheat PP1 proteins allowed us to confirm the strong conservation of the catalytic central core domain and especially the motifs harboring the amino acids involved in metal coordination (GDxHG (Fig 1C), DxVDRG and GNHE (Fig 1D)), in phosphate binding (VDRG and GNHE (Fig 1D)) and in microcystin inhibition docking (SAPNYC (Fig 1E)). More importantly, in all PP1 isoforms identified here the characteristic N-terminal S/T phosphatase domain (Pfam PF16891.14; Fig 1B; [4]) was found highly conserved.

**Fig 2:**
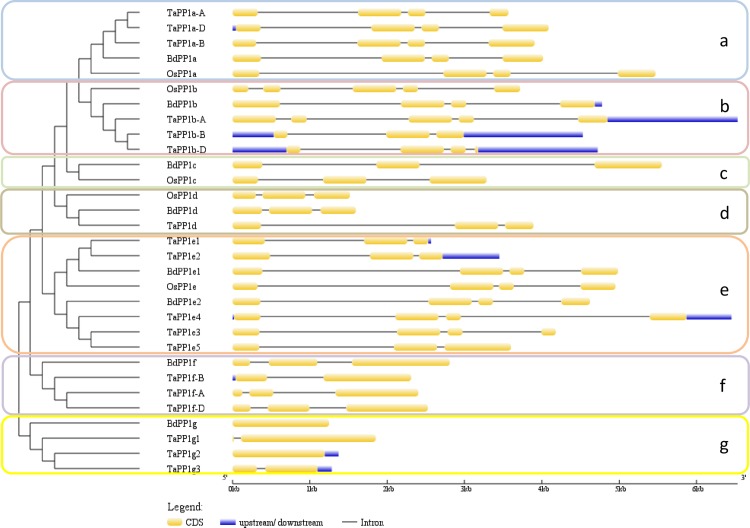
Genomic organizations of the different wheat, Brachypodium and rice genes. cDNA and genomic sequences were downloaded from EnsemblPlant or phytozome and used to draw gene structures with GSDS tool.

Furthermore, the genomic organizations of the respective genes were conserved within each cluster (Fig 2). All these data show that rice, Brachypodium and wheat possess conserved PP1s divided in 5–7 groups. The conservation within each group suggests that despite the redundancy in primary sequence and genomic organization, the distinct isoforms might have each peculiar function.

### Evolution of *PP1* gene family within the three graminea species

The ratio of non-synonymous (Ka) to synonymous nucleotide substitutions (Ks) rates indicates how selective pressure is exerted on genes to conserve or modify protein sequences. The analysis was performed on common wheat, rice and Brachypodium PP1 orthologous gene pairs. The calculated Ka/Ks ratios varied between 0,086 and 0,806 as shown in S3 Table. All the calculated ratios were <1 which means that there are more synonymous than non-synonymous nucleotide changes indicative of a strong protein conservation among orthologs as described earlier by [33] and of a negative selection pressure to conserve PP1 function. These analyses confirm our previous observations indicating that the genes encoding isoforms a, b, c, d of wheat, rice and Brachypodium are more closely related while genes encoding isoforms f and g from wheat and Brachypodium are more divergent.

### Wheat, rice and Brachypodium *PP1* gene expression

To gain more insight into PP1 functions, we investigated the expression of rice, Brachypodium and wheat genes identified herein. For the 5 rice isoforms, previous data described by [12] revealed that *OsPP1d*’s expression is very weak while *OsPP1b* and *OsPP1a* are expressed in the different organs with a higher expression in dividing areas for *OsPP1a*.

**Fig 3:**
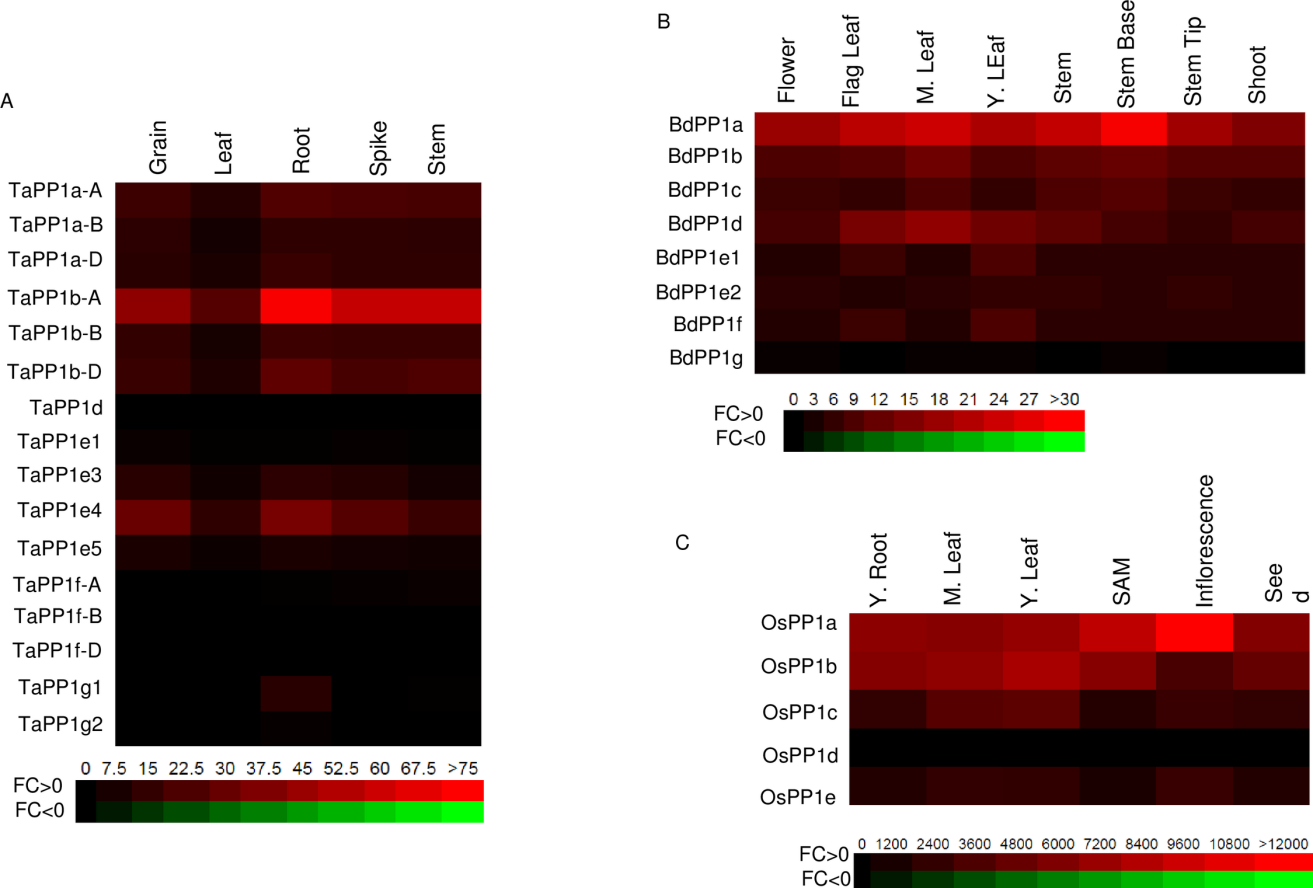
Expression pattern of common wheat (A), Brachypodium (B) and rice (C) *PP1* genes in different plant tissues. The RNA Seq data were downloaded from WheatExpress, BAR and Phytomine respectively. The values are expressed in FPKM. The color-coded scale bar is indicated below each heatmap.

Wheat and Brachypodium *PP1* expression data in the different organs obtained from WheatExpress, Phytomine and BAR are presented in Fig 3. The data were analyzed by comparing the expression of the homeologous genes in the different tissues and comparing the expression of wheat, rice and Brachypodium orthologous genes as member of a same group.

One can observe first that most of wheat and Brachypodium *PP1*s are expressed at various levels. For wheat genes, the member of the b group, *TaPP1b-A* has the highest expression level whatever the plant tissue considered. In contrast, *TaPP1d*, unique member of the d group has the lowest expression level. *TaPP1fs* are expressed at low levels in the different organs studied while *TaPP1g2* is induced in roots. The three homeologous genes of the a group are expressed equivalently in the different tissues whereas within the 5 gene members of the e group, *TaPP1e4* has the highest expression level. Interestingly, the different genes are expressed equivalently in the different tissues indicating an important redundancy. Therefore, one cannot tell which *PP1* isoform would preferentially function in a given plant tissue.

In Brachypodium, the closest *OsPP1a* homolog (*BdPP1a/Bradi1g66970*) has the highest expression while *Bradi2g03597/BdPP1g*, a more divergent PP1 in sequence, is expressed at a very low level. *BdPP1d* is well expressed in contrast to rice and wheat orthologs. Interestingly, rice and Brachypodium group a *PP1*s are mostly expressed in diving areas (inflorescence, stem, young tissues) indicative of a putative particular role in these tissues.

**Fig 4:**
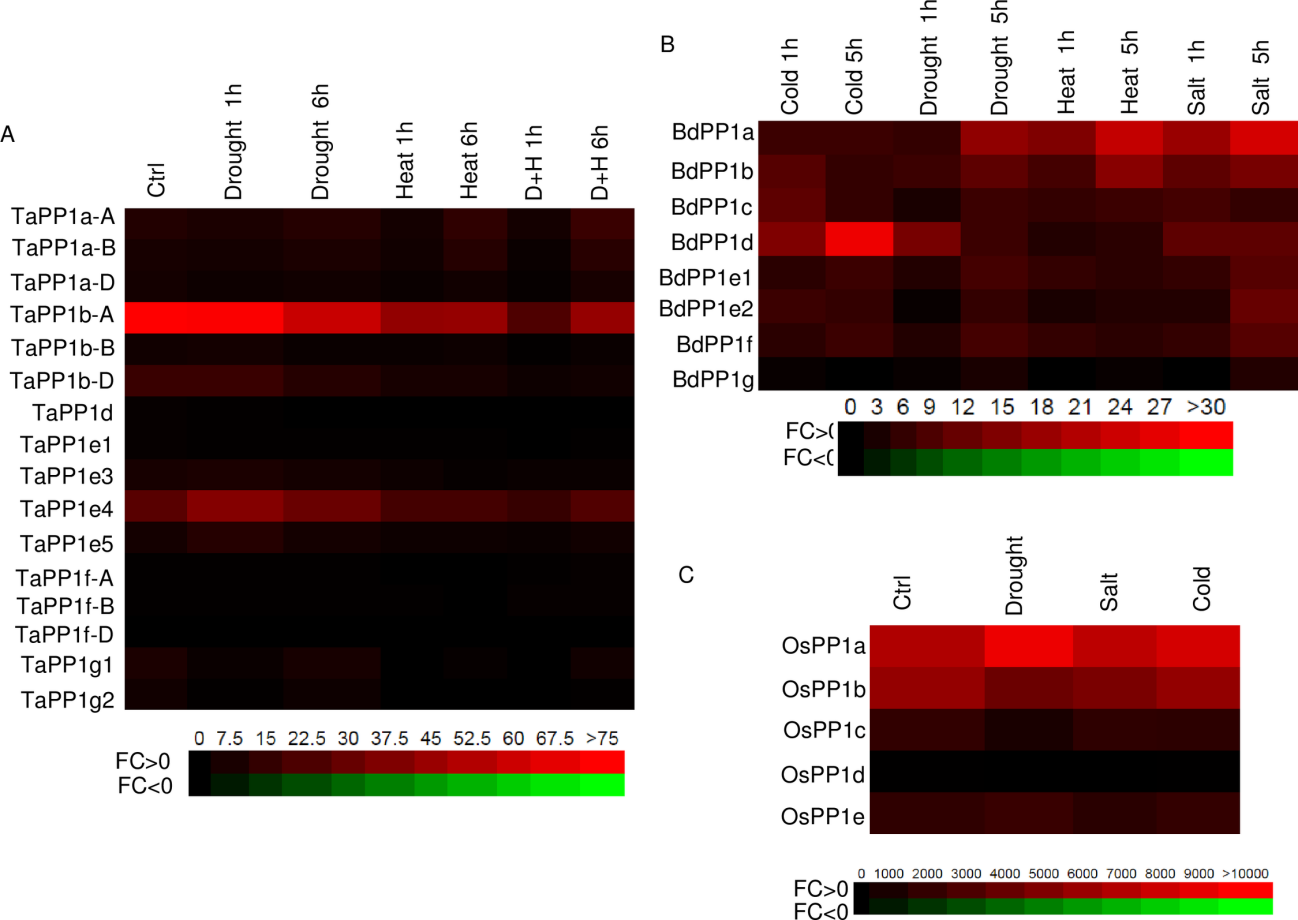
Expression pattern of common wheat (A), Brachypodium (B) and rice (C) *PP1* genes in different conditions. The RNA Seq data were downloaded from WheatExpress, BAR and Phytomine respectively. The values are expressed in FPKM. The color-coded scale bar is indicated below each heatmap.

On another hand, available expression data in response to stress treatments allowed us to observe that the different genes are expressed almost equivalently during stress (Fig 4). The wheat *TaPP1b-A* is slightly repressed upon stress and particularly during a combination of heat and drought stress. Wheat isoforms of the a group are slightly induced after 6h upon the various treatments. *BdPP1d* is strongly induced by cold while *BdPP1a* is induced by salt, heat and drought. *BdPP1e1* and *BdPP1e2* have similar expression patterns and are slightly induced by cold, salt and drought. A similar induction by drought is observed for *TaPP1e4*. However, *TaPP1f* and *TaPP1g* genes are only weakly expressed and not induced by the different treatments applied. No RNA-seq expression data could be found for *TaPP1e2* and *TaPP1g3* suggesting that these genes are either not expressed (pseudo-genes) or very lowly expressed.

The promoters of wheat genes representative of each group were searched for cis-regulatory elements relevant in abiotic stress responses (S4 Table). Cis-elements involved in dehydration responses were occurring more than 10 times (LTRE, ABRE) and others involved in responses to sulfate deficiency (SURECOREATSULTR11) were also found (S4 Table). Rice *PP1* genes are expressed equivalently under different stress treatments. *OsPP1a* is slightly induced by drought and cold while *OsPP1b* is repressed by drought and salt.

### Isolation of *TdPP1a*, a durum wheat protein phosphatase type 1

In order to investigate the involvement of PP1s in wheat stress tolerance and knowing the salinity tolerant phenotype of rice plants over-expressing OsPP1a [23], we decided to focus here on group PP1a and isolate the durum wheat counterpart of OsPP1a from a Tunisian variety. Based on cDNA sequences available for rice, *B*. *distachyon* and mainly *T*. *aestivum*, specific sets of primers were designed to amplify durum wheat PP1 sequences. Using cDNA of Tunisian durum wheat Oum Rabiaa3 variety, a 950 bp fragment could be amplified and cloned. Sequence analyses [24] revealed that the corresponding ORF is highly similar to type one protein phosphatases and encodes a protein of 321 amino acids with a 36 kDa predicted molecular mass. The cDNA was named *TdPP1a* for *Triticum durum* type one protein phosphatase and submitted to GenBank under the accession number KM203893. *TdPP1a* coding sequence, including 5’ and 3’UTR, is 98% identical to *TaPP1a-A*, *TaPP1a-B*, *TaPP1a-D* coding sequences indicating gene duplication in the different subgenomes. Non-synonymous nucleotide substitutions to synonymous nucleotide substitutions ratios show very close evolutionary relationships with common wheat *TaPP1a-A*, *TaPP1-D*, *TaPP1-B* genes (0 to 0.03), with *BdPP1a* (0.032) and *OsPP1a* (0.057) (S3 Table).

**Fig 5:**
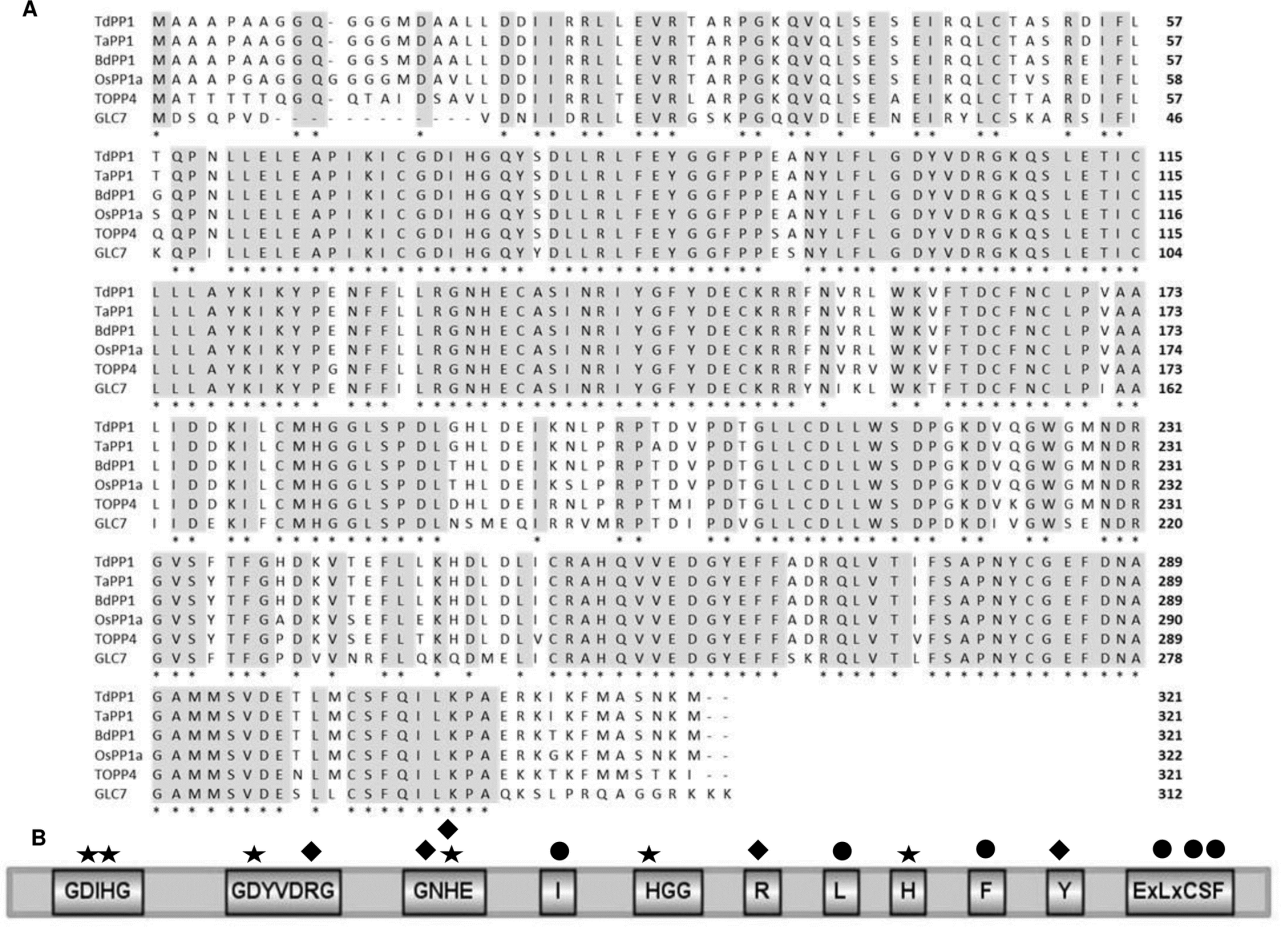
TdPP1a has typical sequence motifs of a type one protein phosphatase. (A) Amino-acid sequence alignments TdPP1a (*Triticum durum* TdPP1a (KM203893) with its orthologs from *Triticum aestivum*. TaPP1 (Traes_4BL_3AA55AD10), *Oryza sativa* OsPP1a (Os03g0268000), *Arabidopsis thaliana* TOPP4 (AT2G39840), *Brachypodium distachyon* BdPP1a (Bradi1g66970), *Saccharomyces cerevisiae* GLC7 (NP_011059) using MultiAlin. Colors indicate: High consensus (dark grey), low consensus (light grey), neutral (white). (B) Structure of TdPP1a showing conserved regions. Diagram was constructed with GPS tool: asterisks and diamonds indicate residues contributing to metal coordination and phosphate binding, respectively. Filled circles indicate the conserved residues involved in binding of regulatory subunits.

TdPP1a protein sequence is identical to the three common wheat PP1a homeologous proteins and is highly homologous to PP1s originating from various origins: 98% with BdPP1a, 96% with OsPP1a, 88% with *A*. *thaliana* TOPP4 and also 77% with bakers’ yeast *GLC7* (Figs 1 and 5). Catalytic and metal binding domains which are essential for PP1 activity [34], are fully conserved (100% identity) (Fig 5). As Ser/Thr phosphatases, TdPP1a and other PP1s harbor also the three conserved residues (Y, W and R) required for the recognition of phospho-sites [34].

In addition, the residues involved in the binding of PP1-interacting proteins forming a hydrophobic groove (where docking of regulatory subunits occurs) are well conserved in PP1s (I, L, F; Fig 5A and 5B) with little differences observed at the ExLxCSF region where most of the PP1s aligned have the ETLMCSF motif except *Arabidopsis* TOPP4 (ENLMCSF) and *S*. *cerevisiae* GLC7 (ESLMCSF). All these alignments confirm the isolation of a typical type one protein phosphatase from durum wheat sharing all the common sequence motifs of PP1s.

Furthermore, the phylogenetic tree presented in Fig 1 shows that OsPP1a is the closest homolog of TdPP1a compared to other OsPP1s (b, c, d, e) that are more distantly related. BLAST searches using TdPP1a as a query on Uniprot database allowed us to identify orthologous proteins from various grasses that form a cluster around TdPP1a and OsPP1a (Fig 1). Compared to *Arabidopsis* TOPPs, TOPP4 is the closest ortholog of TdPP1a and OsPP1a.

### *TdPP1a* expression upon stress treatments

**Fig 6:**
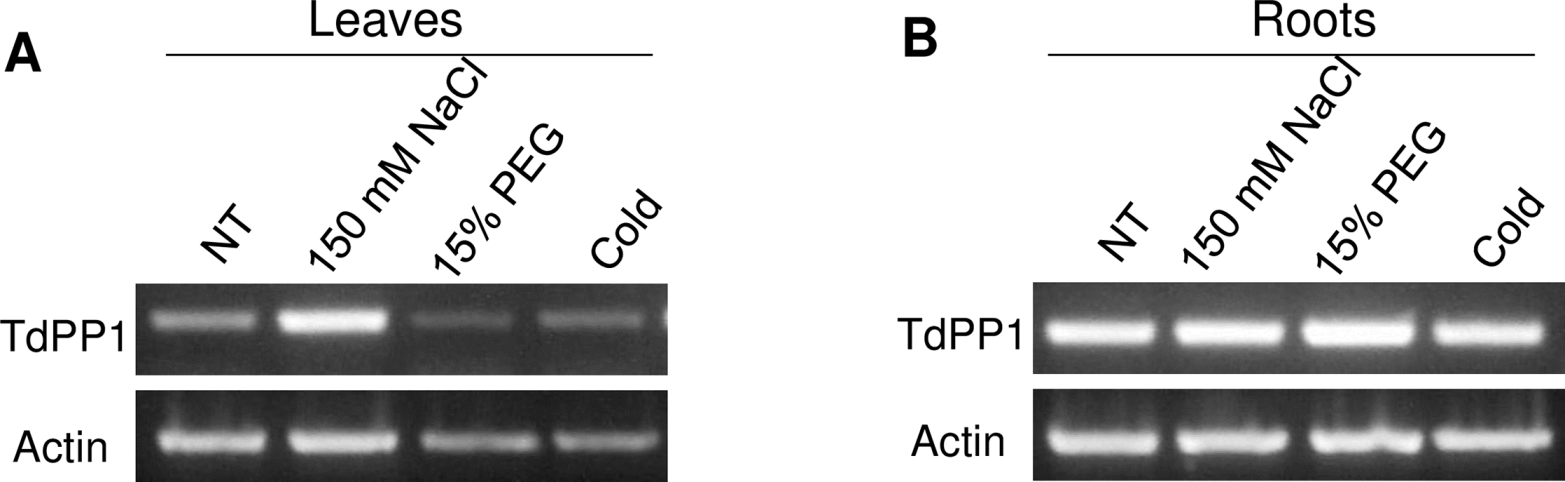
Analyses of *TdPP1a* expression in leaves and roots. 10-day-old durum wheat plants (Om Rabiaa3) were treated for 24 h with 150 mM NaCl, 15% PEG and cold (4°C). *TdPP1a* expression in leaves (A) and roots (B) was evaluated by semi-quantitative RT-PCR analyses. Actin amplification was used as an internal control. All data presented are representative of at least three independent experiments.

In order to examine the role of TdPP1a in wheat stress response, we were interested to monitor its expression under a subset of abiotic stress treatments. For this reason, we treated 10-day-old seedlings of Oum Rabiaa3 durum wheat variety with salt (NaCl 150 mM), cold (4°C) and osmotic stress (polyethylene glycol PEG, 15%) for 24 h. The expression of *TdPP1a* was analyzed by semi-quantitative RT-PCR separately in leaves and roots. As shown in Fig 6, *TdPP1a* was around two fold induced in leaves but not in roots after exposure to salt, when compared to non-treated samples. In contrast, none of other stresses applied induced any changes in *TdPP1a* expression neither in leaves (Fig 6A) nor in roots (Fig 6B). Further expression analyses performed by qRT-PCR confirm the salt induction of TdPP1a, while ABA caused a moderate down-regulation of this phosphatase (S4 Fig). These results suggest that *TdPP1a* is salt induced in leaves and might be involved in wheat salt stress response.

### TdPP1a is ubiquitously localized

**Fig 7:**
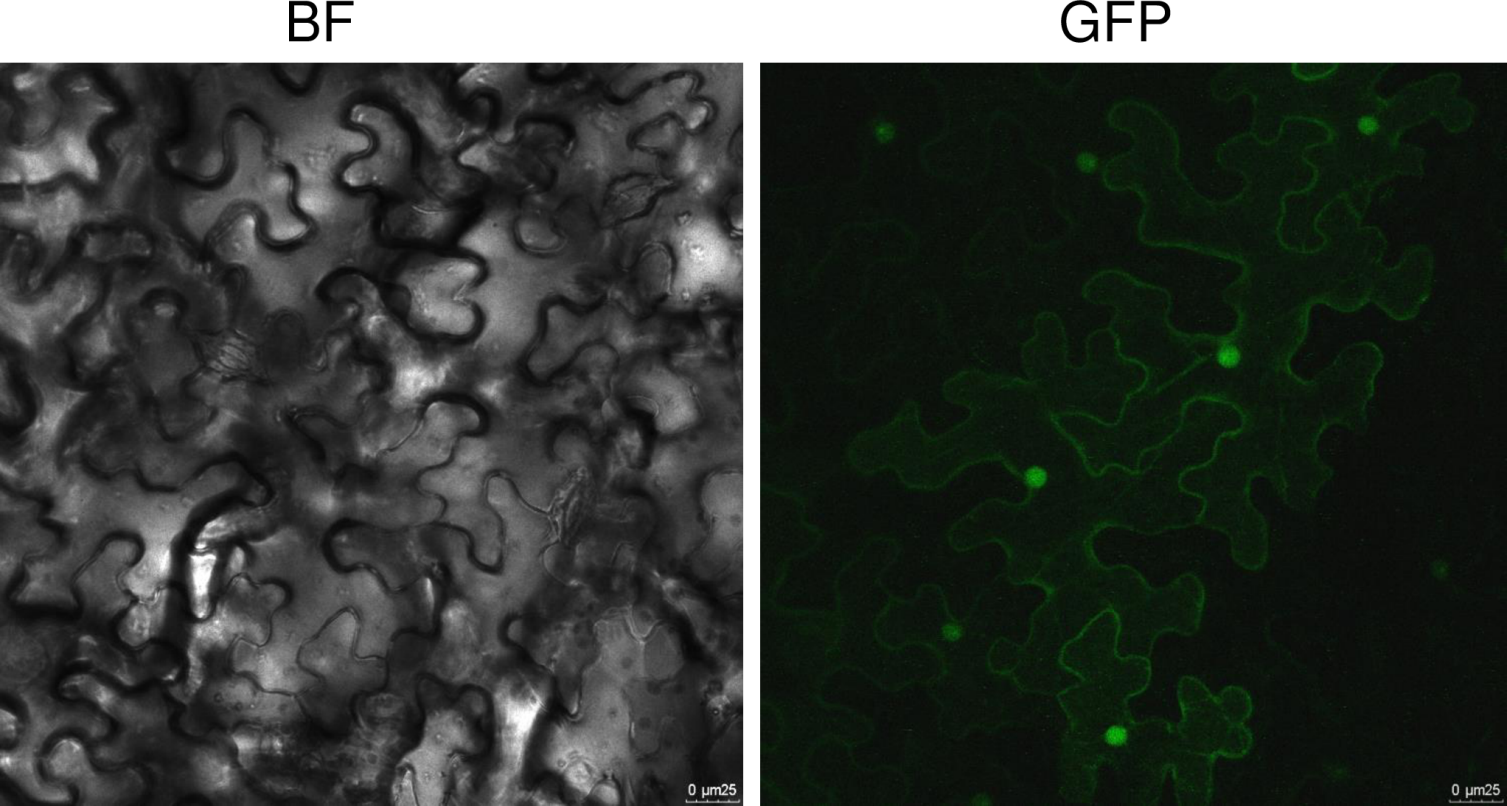
TdPP1a is ubiquitously expressed in *Nicotiana tabacum* epidermal cells. Subcellular localization was investigated in tobacco leaf epidermal cells by confocal microscopy after *Agrobacterium*-mediated transient expression using pB7FWG2-TdPP1a construct. BF, bright field; GFP, GFP fluorescence; TdPP1a-GFP is localized in the nucleus and the cytoplasm. Scale bars = 25 μm.

To investigate the subcellular localization of TdPP1a, we performed transient expression of a green fluorescent protein (GFP)-tagged TdPP1a in *Nicotiana benthamiana* leaves using agro-infiltration. The analysis by confocal laser scanning microscopy revealed that TdPP1a is ubiquitously distributed (Fig 7) with a stronger accumulation in the nucleus. These results are consistent with the localization of TOPP4 [17] and GLC7 of *S*. *cerevisiae* [35] and indicate that PP1s may exert their function at several subcellular locations.

### TdPP1a is an active phosphatase that is enhanced by Mn^2+^ and Fe^2+^

**Fig 8:**
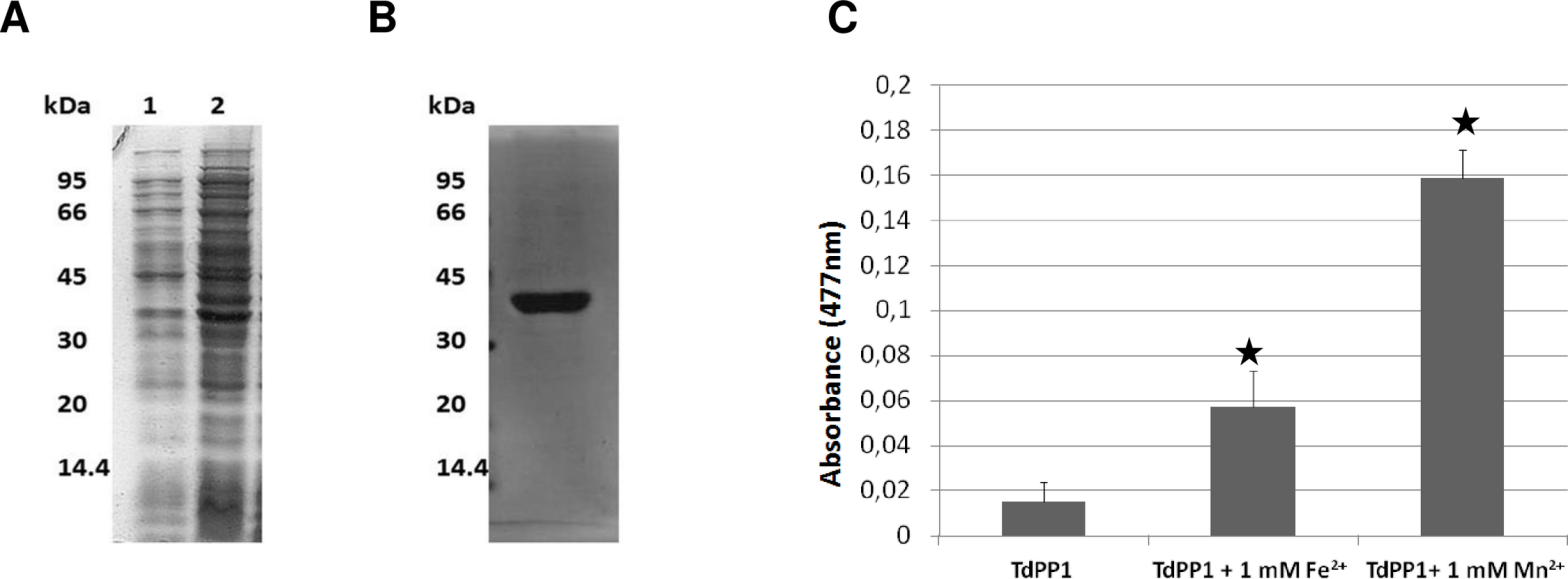
The recombinant His::TdPP1a exhibits an iron/manganese-dependent phosphatase activity. (A) SDS–PAGE analyses of total proteins extracted from non induced (lane 1), IPTG induced (lane 2) bacterial cells expressing 6xHis::TdPP1a. The arrow indicates the induced PP1 product (B) SDS–PAGE after purification of 6xHis::TdPP1a protein on Nickel column. Positions of molecular weight markers are indicated on the left. (C) His::TdPP1a phosphatase activity using OMFP as a substrate. Activities registered on 6xHis::TdPP1a protein in absence or in presence of Mn^2+^ and Fe^2+^ are presented. Values are means of at least 3 independent experiments (± S.E). Stars represent statistical significance (Student’s T-test p<0.01).

To determine whether TdPP1a is an active phosphatase, a His::TdPP1a recombinant protein was produced in *E*. *coli*, and purified on nickel columns (Fig 8A and 8B). Then a phosphatase assay based on the hydrolysis of OMFP was performed at 30°C as previously described ([27, 36]. Knowing that type 1 protein phosphatases are described as metalloproteins and their active site contains putative Mn^2+^ and Fe^2+^ binding sites [1], the phosphatase assays were performed here in the absence and presence of these bivalent cations at a concentration of 1 mM. As shown in Fig 8C, the basal phosphatase activity of TdPP1a registered in absence of any metallic cations is low and increases significantly up to 3.7 and 9.9 folds in the presence of Fe^2+^ and Mn^2+^ respectively. We also tested Ca^2+^, Mg^2+^ and Zn^2+^ that had no significant effect on the catalytic activity of TdPP1a (S2 Fig). Therefore these assays indicate that TdPP1a has a phosphatase activity *in vitro* that is stimulated by Mn^2+^ and Fe^2+^. The stimulation by Mn^2+^ and to a lesser extent by Fe^2+^ strongly suggests the binding of these cations presumably with distinct affinities to specific binding sites (4 His and 2 Asp, Fig 5B) well conserved in TdPP1a and among all PP1s.

### TdPP1a interacts with Inhibitor 2 from Arabidopsis

Inhibitor I2 is a well known regulator and PP1 are characterized by their interaction with I2 [4]. Recently, TOPP1 was shown to participate in ABA signaling by regulating snRK2 activity via an AtI2 interaction [20].

**Fig 9:**
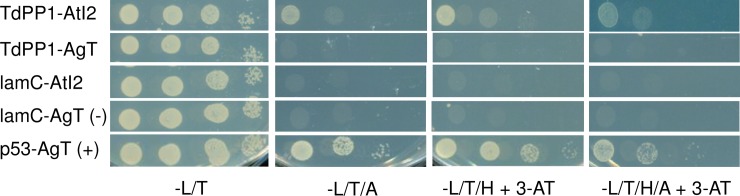
TdPP1a interacts with Inhibitor 2 from Arabidopsis *in vivo*. Yeast-two hybrid pairwise interactions were tested on SD medium lacking Leu and Trp (-LT); Leu, Trp and His (-LTH); Leu, Trp, Ade (-LTA); Leu, Trp, His and Ade (-LTHA) and supplemented or not with 3AT (2 mM). Four independent clones were tested for each co-transformation event. Negative controls used were lamC and AgT. Positive interaction was visualized using the known AgT-p53 interaction. Data shown are representative of three independent experiments.

To see if TdPP1a was able to interact with AtI2 we performed yeast-two-hybrid pairwise interactions, and therefore cloned respectively TdPP1a in fusion with the GAL4 DNA-binding domain and AtI2 with the GAL4 activation domain. Fig 9 shows that AtI2 and TdPP1a co-transformants are able to grow on selective medium lacking Trp, His, Ade and Leu as the positive control AgT-p53 indicating that TdPP1a and AtI2 are able to interact in yeast. In contrast, neither TdPP1a, nor AtI2 when co-transformed with AgT or p53 were able to grow on these media indicating that the interaction is specific. This result indicates that the durum wheat type one protein phosphatase TdPP1a interacts with heterologous AtI2 *in vivo*.

### Complementation of the yeast *glc7* mutation by TdPP1a

The yeast *Saccharomyces cerevisiae* has a single type 1 protein phosphatase encoded by the essential gene GLC7. Different GLC7 mutants were generated and these mutants confer diverse phenotypes varying from cell cycle arrest, resistance to 2-deoxyglucose, increased salt sensitivity, to sporulation defects [37]. The *glc7F256A* (KT2210) mutant is affected in the hydrophobic groove of GLC7 necessary for its binding to regulatory subunits. This mutant is salt sensitive and shows reduced growth at 37°C [31].

**Fig 10:**
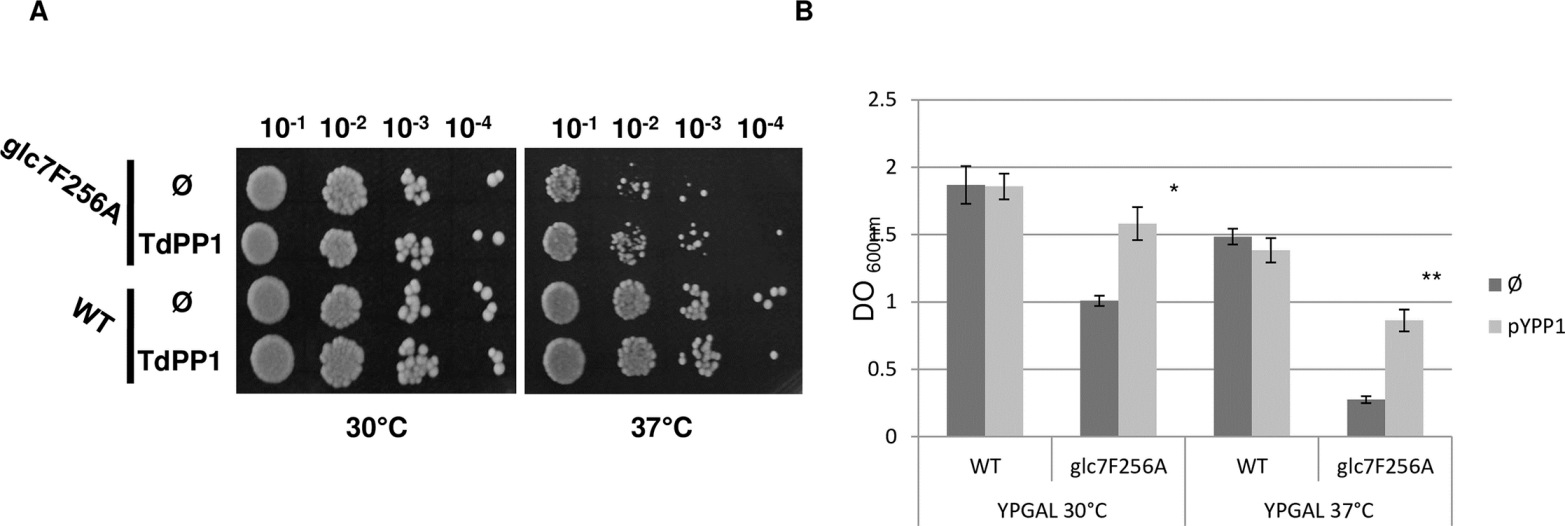
TdPP1a complements *glc7F256A* mutation by restoring growth at 37°C. (A) Wild type and mutant yeast strains transformed with empty vector (Ø) or with TdPP1a were grown for 3 days under normal growth conditions (30°C) or under heat (37°C) in rich solid media containing galactose. Data shown are representative for growth tests of at least three independent replicates. (B) The same strains were cultured in liquid media for 24 h under normal growth conditions (30°C), or under heat (37°C). OD_600nm_ was monitored at 24h. Data presented are means of at least 3 independent experiments ± S.E. Stars indicate statistical differences (**p = 0.0003; *p = 0.015 by Student’s T-test).

To study the functionality of TdPP1a *in vivo* we assessed whether it can complement *glc7* mutation. We cloned *TdPP1a* ORF in the pYES2 expression vector under the control of the GAL1 promoter and transformed *glc7F256A* mutant and wild type strains. Under standard growth conditions (at 30°C), yeast strains expressing TdPP1a and control strains transformed with the empty vector grew equivalently indicating that in absence of stress, TdPP1a does not influence yeast growth (Fig 10A). At 37°C wild type strain transformed with either empty vector or TdPP1a grew slightly less well than at 30°C (Fig 10A). In contrast at 37°C, the *glc7F256A* mutant strain grew more slowly than WT counterpart (Fig 10A) confirming the growth retardation phenotype previously reported by [31]). When expressed in the mutant background, TdPP1a confers albeit modestly, higher growth rates at these non permissive temperatures, compared to the control strain transformed with the empty vector.

To have a quantitative measure of the growth, we did the same experiment in liquid culture where we monitored growth by optical density absorbance (OD_600_) after 24 hours of growth in rich media containing galactose as carbon source. The data presented in Fig 10B show that wild type strains expressing TdPP1a or not, grew equivalently well at permissive temperatures. In contrast and more interestingly, the growth of *glc7F256A* was significantly improved when TdPP1a is expressed. Indeed, Fig 10B shows that the growth of *glc7F256A* cells expressing TdPP1a is significantly improved by around 33% at 30°C compared to the strain transformed with the empty vector. Moreover, at non permissive temperatures, *glc7F256A* transformed with the empty vector barely grew while the same mutant strain transformed with the TdPP1a construct showed clearly a significant increase in growth. The growth was indeed improved by more than 60% indicating that the expression of TdPP1a is able to restore to some extent the growth arrest of *glc7F256A* at 37°C. However, the growth rate at 37°C of *glc7F256A*::TdPP1a is still lower than that of wild type strain. All these findings indicate that TdPP1a partially complements the *glc7* thermo-sensitivity and that TdPP1 acts as type 1 protein phosphatase *in vivo*.

## Discussion

In the current work, we report the identification of type one protein phosphatases from wheat and Brachypodium and detail the different isoforms existing in these plants compared to the known rice PP1s. We show that Brachypodium and wheat contain more PP1 genes than rice but they mostly cluster with the 5 rice PP1 isoforms indicating that grasses harbor distinct isoforms that may have distinct functions. Within each cluster the genomic organization is conserved as well as the protein primary sequence. The difference in the different isoforms lies in the N-terminal domains that are variable in sequences and in size as described earlier ([4, 34].

The *in silico* expression studies performed here revealed that among orthologous isoforms, the overall expression is conserved but the fact that the expression of an isoform is not restricted to a particular tissue or treatment strongly suggest functional redundancies.

However, knowing that type one protein phosphatases activities are controlled by specific binding of positive and negative regulators, this functional redundancy might be circumvented by the specific binding of regulatory proteins.

The durum wheat TdPP1a isolated from the Tunisian variety Oum Rabiaa3, is the first wheat PP1 cloned so far. We show that TdPP1a has a conserved primary protein structure highly similar to other PP1s from cereals, dicots but also to mammalian and yeast PP1s. The domains important for the catalytic activity, metal ions and regulatory subunits binding are highly similar indicating that among eukaryotes the well conserved PP1 primary sequence may account for conservation in tertiary structure but also in function.

A recombinant form of TdPP1a was produced in *E*. *coli* and *in vitro* assays showed that TdPP1a has a weak phosphatase activity which can be strongly enhanced by iron or manganese indicating that TdPP1a is a typical metallo-dependent, type one protein phosphatase as described for mammalian PP1 [38, 39]. Although *in vitro* TdPP1a has a weak phosphatase activity, this activity might reach higher or even lower levels *in vivo* by the binding of PIPs.

One of the best characterized PIP is ‘Inhibitor 2 (I2)’ first characterized as a functional catalytic inhibitor of PP1 in mammals [40] but also as PIP in plants [20]. Type one protein phosphatases have also been characterized by their specific interaction with I2 [4]. Herein, we could show that TdPP1a is a typical PP1 since we show that PP1 is able to interact with heterologous I2 from Arabidospis in yeast-two-hybrid system.

To demonstrate that TdPP1a is functional *in vivo*, we showed that once expressed in the yeast *glc7F256A* mutant strain, the wheat phosphatase can restore its growth deficiency at non permissive temperatures. The complementation is partial since the mutant strain overexpressing TdPP1a does not grow at an equivalent rate as registered for the wild-type strain. Previously, *glc7* complementation studies using TOPP1, TOPP2 and TOPP3 revealed that only TOPP2 could complement the yeast *glc7* mutation [41] indicating that the different TOPPs might not be functionally redundant. Therefore, despite the overall high sequence conservation small differences still persist between these phosphatases and may impact their functionality. Moreover, one cannot exclude that expression of TOPPs or TdPP1a at high levels might be deleterious to yeast cells as it was previously observed with GLC7 overexpression [42], hence hindering full functional complementation. This hypothesis is reinforced by the fact that we failed to generate transformants in *glc7* mutant background with a construct harboring *TdPP1a* under the control of a constitutive promoter. Such assumption suggests that constitutive expression of TdPP1a may be lethal in yeast.

BLAST searches indicate that the wheat PP1 isoform identified here is phylogenetically close to OsPP1a from rice. It is worth mentioning that OsPP1a was previously reported to interact with RSS1, a crucial regulator ensuring meristem maintenance under salt stress, possibly by regulating negatively the rice OsPP1a phosphatase activity [12, 22]. Hence, PP1 inactivated, its well known substrate in dividing cells, pRB, stays hyperphosphorylated stimulating cell division [22]. The close homology between TdPP1a and OsPP1a may indicate that they both function in similar regulatory pathways. The identification of the wheat homolog of RSS1, TdRL1, indicates the existence of the RSS1-like pathway in wheat [43] where TdPP1a could be regulated by TdRL1. However, the redundancy between the different wheat and rice PP1 isoforms and the fact that all OsPP1 seem to bind, although weakly to RSS1 [12, 22], suggest a possible flexibility in the interactions between RSS1 and PP1s depending perhaps on tissue and/or environmental conditions. Interestingly though, OsPP1a when over-expressed in rice alleviates salinity stress indicating that it exerts a positive effect in stress tolerance [23] contrasting with its suggested role in the RSS1 pathway [22].

In this study, we show that *TdPP1a* is expressed equally under normal conditions in roots and leaves as observed for other *PP1*s [12, 13, 17]. Importantly, an up-regulation of *TdPP1a* was observed in leaves upon salt stress (NaCl 150 mM) while the expression remains unchanged in roots under the same conditions. These data suggest a specific role of *TdPP1a* during salt stress in the aerial parts of the plants which might not exist in the roots. *TOPP1’s* expression is also slightly induced in leaves after 3h of salt treatment (data from ePlant/BAR) but the nine *Arabidopsis TOPPs* have similar expression patterns independently of plant growth conditions, hormonal treatments and plant tissue (data from ePlant/BAR). In the case of *Vicia faba*, *VfPP1c-1* seems to be specifically expressed in guard cells while *VfPP1c-2*, *3*, *4* are not expressed there. Yet, VfPP1c-1 promotes stomata opening upon blue light signaling [13]. The expression landscape of *PP1* transcriptional regulation in the different wheat tissues and under specific conditions described here accounts for this redundancy. However, because PP1s’ activities are modulated by PIPs and are inactive solely, transcriptional regulation might not be decisive in the regulation of PP1s’ function. This is suggested by the fact that, in Arabidopsis, stress responses controlled by ABA are negatively regulated by TOPP1 and AtI2 [20] and that TOPP4 positively regulates GA signaling [17] but neither *TOPP1* nor *TOPP4* expression is regulated by ABA or GA, respectively (data from ePlant/BAR). In our case we noticed a moderate down-regulation by ABA of *TdPP1a* in wheat aerial parts (S4 Fig). Nevertheless, the role of TdPP1a in regulating ABA responses remains to be investigated.

Confocal analysis showed that *TdPP1a* is like TOPP1 and TOPP4, ubiquitously expressed within the cell under normal conditions, but seems to accumulate preferentially in the nucleus. This observation suggests that TdPP1a phosphatase activity might be cytoplasmic and/or nuclear [17, 20] and that relocation might occur in a PIP-binding dependent manner.

The fact that plant cells have various PP1 isoforms that are highly conserved in structure, expressed at similar levels in different tissues and under similar conditions, renders the comprehension of their regulation mechanisms more difficult and likewise their roles in specific signaling pathways. Indeed, in *Arabidopsis*, insertional *topp1* and *topp4* mutants exhibit little phenotypic changes hindering researchers to decipher their functions [17, 20]. The yeast GLC7 multifunctionality accounts also for a particular role of PIPs in specifying GLC7 functions within the cell depending on the cell cycle phase and the environmental conditions [5, 6]. Thorough functional characterization of these plant phosphatases and their specific PIPs, will certainly help to unravel the complex network of functions involving PP1s to gain more insight in the importance of the reversible phosphorylation processes driven by PP1s, especially in modulating plant stress responses.

## Supporting information

S1 Fig cDNA sequences of Brachypodium and wheat PP1 genes. identified downloaded from phytozome and EnsemblPant.
(DOCX)

S2 Fig Phosphatase activity measurements. Recombinant His::PP1a phosphatase activity using OMFP as a substrate. Activities registered on 6xHis::TdPP1a protein in absence or in presence of Ca^2+^, Mg^2+^, Zn^2+^, Mn^2+^ and Fe^2+^ (1 mM each). Values are means of at least 3 independent experiments (± S.E). Stars represent statistical significance (Student’s T-test p<0.01).
(TIF)

S3 Fig Phylogenetic analysis based on wheat, Arabidopsis, rice, Barachypodium cDNAs. Phylogenetic tree was obtained using MEGA6 with the Neighbor-Joining method based on cDNA sequences from EnsemblPlant, phytozome and TAIR. Wheat sequences are indicated with red dots, rice with light blue squares, Brachypodium PP1 genes with green diamonds and Arabidopsis with dark blue triangle. The percentage of replicate trees (bootstrap test with 1000 replicates) in which each taxa clustered together is indicated on each branch. The tree is drawn to scale and indicates evolutionary distances in number of synonymous to non-synonymous nucleotide substitutions.
(TIF)

S4 Fig Relative expression of TdPP1. Seven-day-old durum wheat seedlings were treated with NaCl (150 mM), or ABA (100 μM) for 1 hour. (A) Relative expression of TdPP1a was analyzed by quantitative real-time qPCR using wheat actin as control. (B) Raw data of relative expression (RE) with standard error (SE).
(TIF)

S1 Table PP1 wheat genes and their orthologs of *T*.*urartu* and *A*. *tauschii*. (DOCX)

S2 Table Wheat, Brachypodium and rice PP1 proteins. Accession number, size, molecular weight, pI, instability index and GRAVY. The latter prarameters were predicted with protparam.
(DOCX)

S3 Table Evolutionary pairwise distances between wheat, Brachypodium, and rice PP1s by calculation of the Ka/Ks ratios. Ka/Ks ratios were calculated using MEGA6.06.
(DOCX)

S4 Table Most relevant cis-elements found in promoters of representative wheat genes identified using PlantPAN2.0. (DOCX)
