# Supplementary material for: Genome wide identification of wheat and Brachypodium type one protein phosphatases and functional characterization of durum wheat TdPP1a
Source: PLoS One. 2018 Jan 16;13(1):e0191272. doi: 10.1371/journal.pone.0191272 (PMC5770040; doi:10.1371/journal.pone.0191272)
Supplement: S1 Fig — identified downloaded from phytozome and EnsemblPant. (DOCX) [file pone.0191272.s001.docx]

>Bradi1g00690 Org_Bdistachyon transcript: Bradi1g00690.1.p myosin phosphatase activity (Blast2GO) (PAC:32805800)

ATTCGGGCGCGGCAGCAGCAGTATTCGCTATCGCCCCCACTTTAAATCTGTGCCGACTGACCTCGCTTTCTTGCCGTGGCAGCTGGTGCAGTCTGCATCATCTCTCTGGAATTGCTTGGTTCTTCTTCGCCTAGTCTCCTCTCCTTTCCCGGCCGGCCGGACCGCATTTGCTTGCACTTTGATACCTCGATCCTTTGCTGCGCGAGTGGTCCTTGAAGCTCAAAGGAAAACGGAATCTTGCATTTGTGGTGGGAGGGAGGAGAAAGGGGGGAATCTTATCAAAGCAGCCTACCACTGAGATTTGATTGGCGATCGCTGTATATATAGCTTATCCTTCCATTCCTTTTCTGAAGCAGCTGCTGCCTAGCGAGCAGCACGTACGCCCTGTACTCTGCTCCACAACAGAATAGGATGCTCCTAGCTAGCAAAATCTTCTCTTGCTCGGTCGGCCTTCTCTGATGTGGCCTTCGATTGATGCAAAGTTATGCTAGCTAGGTTACTAATATAGGCTGGCTCGTCGACTTCTGGTCGTCTACTTAAGCGTCTGCAGTTCTTCGTACTTGTTGTCACCCCGGCCGGCCCATAAGTACTGGACAATCAGTCCTGCAACTGGAGATGCCTAGCTAGAGCTCTTATTTATTCGATTTAGTTAATCAGGTAGTAAGGTCGATCATGGAGCGTGCGGCGCTGGACGACGTGATCCGGCGTCTCCTTGAGGTGCGGCGGTACCGGGCAGGGAAGCAGGTGCAGCTGGCGGAGTGGGAGATCCGGCAGCTCTGCGCCGCCTCCAAGGACGTGCTCATGCGCCAGCCCAACCTCCTCGAGCTCCAAGCGCCCATCAAGATCGCCGGCGACATCCACGGGCAGTACCCGGACCTGCTGCGGCTGTTCGAACTCGGCGGGTTCCCGCCAAAGCACAAGTACCTGTTCCTGGGCGACTACGTGGACAGGGGAAAACAGAGCATCGAGACCATGTGCCTGCTGCTGGCCTACAAGCTCAAGTACCCGGAGCACTTCTTCCTCCTCCGGGGCAACCACGAGTGCGCCTCCATCAGCCGCGTCTACGGCTTCTACGACGAGTGCAAGCGCCGCTACTCCGTCCGCCTCTGGCGCCTCTTCGCCGACGCCGTCTTCGCCCACCTCCCGCCCGCCGCCCTCGTCGACGGCCGCATCCTCTGCGTCCACGGCGGCCTCTCCCCGCAACTCCTCCTCCGCGCCTGCGACCTCCGCCGCGACATCAACGCGCTCCCCAGGGTGCCAGACGTCCCCGAGTCCGGGCTCCTCTGCGACCTCCTCTGGTCCGACCCAGCCGCCGCCGCCGGAGCCGGAGGGGCTTCGGCTTCTTCCGGGTGGGGGGAGAACGAGAGGGGCGTGTCCTACACGTTTGGGGCGGACGTCGTGGAGCGGTTCCTGGAGCGGCATGACCTCGACCTCGTGTGCCGCGCGCACCAGGTGGTGGAGGACGGCTACCAGTTCTTCGCCGGGAAGAGGATGGTCACCGTCTTCTCCGCGCCCAATTACTGCGGCGAGTTCGATAATGCTGCCGCCATCATGTGCGTCGATGATGAGCTCACTTGCTCGTTTCAGATCATCAGGCCTGCTTCTGCTCGTTACAGCACCAGCAGCAGAGGGATCCGGTCGCCCTGGTGTTAACTAGCTGTATGTTGAGCTCGATTTTCTTCAGTTGGGCTGAATCAGTGTAATAATTAATCGGATTTGATTTTGGTCTTTGTAATGTACTAACGGCGATTTCAGAGGGGATAGTCGGAACTAGACCGATTACTTTTTTTTTACGGATTATCATGTTGGGTTGGGCCGACATCTCTTTGGTCAAAGAAGAAGAAGAAGACGGTCTACGCGGGCCGGGTATGGGCGTATGGCAGAAACTGGACCAAGACGAGAGAGGAGGAAGAAAAAAGATCTTCTATAATTGGGCCTCATGCATTCCAGCACTACCGATCGCGCCTTCATCTAGTGGCCACATACGTATTGGGCCACGGCCCGACATCTCCCGAAAAAGAACATTCATATGGCTCTAAAAAAAAAACATACCGGGTGATGCTTCAAAAAAACGGATATTGCTTCATGTTACTCTAATTTATTAATTTAACCTT

>Bradi1g48410 Org_Bdistachyon transcript: Bradi1g48410.1.p myosin phosphatase activity (Blast2GO) (PAC:32806122)

TCCGCGCCCCTCTCTCTGTCTACGCATTTCCGTTTTTTCTTTCCTCCCCTTTCTCTCTCTTCCCACCCCCCAGCCGTCGGCGGAAGGAAAAAGAAAGGCACGGCTAGGGTTTAGCCGCCGCCCCCGCTTAGCCTTGCCTCCCTCCCCGCAGCCGCCAGGGTTTTACCTCCCTCCGTCCCTTTACCGAGGCGCCGGGCGATGGCGGCGATGGACGCGGAGACGCTCGACGACGTGATCCGGCGGCTCCTGGAGGCGCGCGGCGGCCGCACGGCGCGCCCCGCGCAGCTCTCCGACGCGGAGATCCGCCGGCTCTGCGCCGCCGCCAGGGAGGTCTTCCTCGGCCAGCCCAACCTCCTCGAGCTCGAGGCCCCCATCAAAGTCTGCGGGGACATCCATGGACAATATACTGATCTTCTTCGATTATTTGAGTATGGTGGCTTCCCACCGGAGGCAAATTATCTGTTTCTGGGAGACTATGTTGATCGTGGCAAACAGAGTATTGAAACAATATGCCTTCTACTTGCTTACAAGATAAAGTACCCGGAGAACTTCTTTCTCCTTAGGGGAAACCATGAATGTGCGTCGATCAATCGAATTTATGGATTCTTTGATGAATGCAAGAGGAGGTTTAACGTTCGTCTCTGGAAGGTTTTTACTGATTGTTTTAACTGTCTCCCTGTGGCTGCTCTTATTGATGATAAGATCCTGTGCATGCATGGGGGTTTATCACCTGAGTTAAAGAATATGGACCAGATACGCAACATTGCCCGTCCTGTGGATGTTCCGGACCATGGTCTCCTCTGTGATCTGCTGTGGTCAGACCCAGACAAGGAAATTGATGGCTGGGGTGAGAATGACAGGGGTGTGTCTTACACATTCGGAGCTGATAAGGTTGCGGAGTTCCTTGAGAAACATGATCTCGATTTGGTCTGCAGAGCCCACCAGGTGGTGGAGGATGGGTATGAATTCTTTGCTAAGCGCCAGCTTGTAACCATATTCTCGGCACCCAACTACTGTGGCGAGTTTGACAATGCTGGTGCTATGATGAGCATAGACGATTCCTTAACATGCTCATTCCAAATTCTTAAGCCTTCTGATAAGAAAGGAAAAGCTGGAGCTGGCAACATGTCAAAACCTGGAACGCCTCCTAGGAAGATAAAGATTAATATTATTTAGTCAGATTCTGTCCGATATGAACTGAGTGATTGTGATGGAGCACCCCCAAACTTGTTATGGTGAGATTTTAAGGTGCACTGAACATCATGTCGAAATGGCAAACTGCGTGCCTTTGTCTTTCACGGTCTGATGCAGATCTGTCCTGCCATTCTGAAAACTTCGTACTTCGGATCAGAAAACCATAACTGGTTAGCTCTTTTTATGTGGATTTTTTATTCGCTCTTACTCCTGCACAAGTCAAAATGTTCGTGCAAAGTGAGGTATCGGGAACATGATCATGCGGTGGAGATCGCTGGTCTTAAATGTAGATTTATGTTTTGTATGCTGTTTTATCTGTAAAGCTTGCCTTTGAAGATTATACTGACCAGCTTTAGTGGATGGCTTTCACTGATGCTGATGGTAGAGAATGACTGGTATTTATGCCCAAATTGCTGGCAGTTTTTTCCTTGTGGTAGGACAACGCTTTTGTGTTGCTGTTTTAAACAGTTTTAGGTTTGCCACATTATGTGATGTTTTTTATGGTTCACTGGGTATTGAAGTGAAACATGCCATTGATTATCCCTTGAGGCATGAACTCACGGTATCATTTCATTTGCACTGAATGGAGGCTACCTCACTGGTGGTCACATAAATTTGTC

>Bradi1g66970 Org_Bdistachyon transcript: Bradi1g66970.2.p NF-kappaB-inducing kinase activity, myosin phosphatase activity (Blast2GO) (PAC:32803178)

AGGAGTAAAACAAACAAAACTCAAAGGACGAGACAGGTTTATCTAGTACCTATCGGCGGCCCACCACCCAATCTCCCACCCCCTCGGCCACCTGCTCCGCCCGAAGCGCCGAAACCCTAACTCCACCGATCTCTCCAGCCCGTGGGGAGGCGATGGCGGCGGCGCCGGCGGCGGGAGGGCAGGGGGGCAGCATGGACGCCGCGCTCCTCGACGACATCATCCGCCGCCTGCTCGAGGTGCGGACGGCGCGCCCCGGGAAGCAGGTGCAGCTCTCCGAGTCGGAGATCCGCCAGCTCTGCACCGCCTCCCGCGAAATCTTCCTCGGCCAGCCCAACCTCCTCGAGCTCGAGGCTCCCATCAAGATCTGCGGTGATATCCATGGTCAGTACAGTGATCTTTTAAGGCTCTTTGAATATGGAGGTTTTCCCCCAGAGGCCAATTATCTATTCTTAGGTGATTATGTTGACCGAGGCAAACAAAGCCTGGAAACTATATGTCTCCTTCTTGCATACAAAATCAAGTACCCGGAGAACTTTTTTCTTCTGAGAGGGAATCATGAGTGTGCTTCAATAAACAGAATATATGGATTTTATGATGAATGCAAGCGTCGATTCAATGTGAGGTTATGGAAGGTTTTCACGGACTGTTTTAACTGCCTCCCCGTGGCTGCTCTAATTGATGATAAAATATTATGCATGCATGGCGGCCTTTCTCCTGATCTGACACATTTGGATGAGATAAAAAACTTGCCCCGTCCTACTGATGTGCCGGATACTGGTCTACTCTGCGATCTTCTTTGGTCTGATCCAGGAAAAGATGTTCAAGGGTGGGGCATGAACGATAGGGGGGTCTCATACACATTTGGCCACGACAAAGTAACGGAATTCCTTCTAAAGCATGATCTTGATCTTATTTGCCGGGCTCACCAGGTTGTTGAGGATGGTTATGAATTCTTCGCTGACAGACAACTGGTCACCATCTTCTCAGCTCCCAATTATTGTGGTGAATTTGATAATGCTGGCGCGATGATGAGTGTTGATGAAACTTTGATGTGTTCTTTTCAAATTCTCAAACCTGCTGAGAGAAAAACAAAATTTATGGCGTCAAACAAAATGTGAAATGGTGAAGTTCTTTCCTCTTCTAGGCTCTGAGGCGCTCTCATGCGGACACAATCACATCAAGACATCAACATGCAGGCGCTAGGCTTGAAGGCAGCATCGGGCTAATTCTACTGGGGAAACCAAATAATATCATATCTAACAGTTGGTCTGAAAGTTACTCTTCATAGCAAGTGCCAGATTTTTTATCCTTTTCTGTTACTGTGTGCGGAACCATCTTGTAGCTGTAGATATATTGATTATGGAAGGATGAAGTTGCGAGTTGACCTCAGGTGATTTCCTTCACCAGCAAGGAGAAATTCTGAATATGAAAGTATCTGTTTGGAAGTTCCACTATTTAAAGATCACCATGTATCACTTTATGAAGGTAAGAATGCAAGCCAAGGCCGTGTTTCTCAGTTGCTTGTAACCAAAGTTTAGATAGATGAATTTTATCTTAGATTCTAGGGAACTCCAATGGCAACAATTTGATTAGTGCGAGCACACGGAATGCTGACGCTTCGTGAGAAACTTGCCGAAGACACCTTGCAAGAGGCTAGATGGCTTGAAATTTATT

>Bradi2g03597 Org_Bdistachyon transcript: Bradi2g03597.1.p (M=4) PTHR11668:SF280 - SERINE/THREONINE-PROTEIN PHOSPHATASE PP1 ISOZYME 7 (PAC:32776942)

CAATTCCGAGTCGTGTGGGAGTTGGACTCCGCCGTTATATATGACCCGACGATCGATTCTATCAACAAAATAACACGAAATCGATCGATCATGGAAGGAGATGCCGGAGCGCCGGCGCTAGACCTCGACGGGATAATCCACCGCCTGACCACAACCACCTCCGCACCCCCAAAACCTCCCCCAGTAACCCGCGCCGAAATCAAAGCCCTCTGCGCCGCCGCGAAGCAGCTCCTCCTCGCCCAACCGATCCTCCTCGACCTCCCCGCCCCCATCAACATCTGCGGCGACATCCACGGCCAGTTCCCCGACCTCCTCCGCCTCTTCTCCCCCGCCGTCGGGGGCCCGCCGTCGCCCGCCAACCGCTACCTCTTCCTCGGCGACTACGTCGACCGGGGCACCCAGAGCGTGGAGACCGTCTGCCTGCTGCTGGCGTACAAGCTCAAGCACCCCGACGCCTTCTTCCTCCTCCGCGGCAACCACGAGTGCGCCGCCATCAACAAGGCCTACGGCTTCCACCAGGAGTGCGTCGACCGCCGCCTTATCATCCCCGGGCGGTGGGACTGCTGGACGGAATTCAACCTCGTCTTCGCCTGCCTCCCCCTCGCCGCCCTCGTCGCAGATCGGAAGAATAAGAAGAAGATGTTCTGCGTGCACGGGGGGCTGTCGCCGGAGCTGGAGACGATGGGCCAGATCCGCGCGCTCCGGCGGCCGCTGCCCGTCGAGGTCCCCGAGAAGGGGCTCCTCTGCGACCTGCTGTGGTCCGACCCCGCCGCCGACGAGGACGACTGGGGATGGGGCGAGCCGCGGCGGGGCGTCTCGTCGTGCAGCTACGGCGCCGACGTCGTGGCGGAGTTCTGCGAGCGGCACGGGGTGGAGTTGGTGTGCCGGGCGCACGAGGAGAAGCAGGCGGGGTACGAGGTCGCCGCGGCGGGGAAGCTCGTCACCGTCTTCTCGGCGCCCGACTACTGCGGCACAACCGGGAATGACGGCGCCGTCATGGTTGTCGACGGCGCGCTCGCGTGCTCCTTCCGTGTTATCAAGCCTGCTGCTCGTCCCGCCGGGTCGTTCCTCGTGCTCGACCTCGATCCCAACGCTCCTCCCGTTGCTCATGAGGATTAGTCGTCCTTGAACACTGATTCTGTTTTAGATTTTATCTACTCTCTTCTTTGATTTTTGATTCTTGAATATACTTGTGCTTGTTCCGTTGTTGTTCATGGTAATGGAAGTTCTTCAAACAGGGATACGCAAG

>Bradi2g12650 Org_Bdistachyon transcript: Bradi2g12650.1.p NF-kappaB-inducing kinase activity, myosin phosphatase activity (Blast2GO) (PAC:32782391)

CACATGGACGGAGCCAAAGAAACATCCAGCCGTCTGTCCTGGGTCCGACAGTGGATCTTCCTTGACGCGCTTTCGATCACTTTGGCCCACGCCATTATCCAATAATCCCCACGCCACCCTCCATTTCTATTCCTCCTTTTCCGTCTCCGTCGCCGTCGAGGGACCTCAGGGGAAGAAAGAACAAGATGATGATGACACGGGCGTCAATGGGGGCCATGGACGGGGCGGCGTTGGACGAGGTGGTGCGGCGGCTAGTCGAGGGAGGCCGCGGGGGTCGCCAGGTGCAGCTCTCGGAGGCGGAGATCCGCCAGCTCTGCGTCGAGGCCAAGAGGGTCCTCCTCTCGCAGCCCAACCTCCTGCGCATCCACGCGCCCGTCAAGATCTGTGGTGATATCCACGGTCAGTTTGTTGATCTTCTGAGGCTGTTCGATTTAGGTGGCTATCCTCCAGCTTCAACTTATCTTTTCCTTGGAGACTACGTGGATAGAGGCAAACAAAGTTTGGAAACCATATGTCTGCTTCTGGCATATAAAGTGAAGTACCCTGATAAGGTTTTCCTGTTAAGGGGAAACCACGAAGATGCAAAAATTAACAGAGTTTATGGTTTCTATGATGAATGCAAGAGGAGATTCAATGTTCGTCTGTGGAAGATATTCTGTGATTGCTTCAACTGCTTGCCTATGGCAGCACTTATTGATGACAAGATATTCTGCATGCATGGTGGCCTCTCACCTGAATTGAATAGCTTAGATCAAATAAAGGATATCGAGAGGCCTATTGAAATTCCTGACTACGGTCTTCTGTGTGATTTGCTTTGGTCTGATCCTAGTGCTGACACACAAGGGTGGGGGGAGAGTGATAGAGGTGTTGCTTGCACTTTCGGTGCAGATAAGCTTGTAGAGTTTTTGGAGAAGAATGATCTTGACCTTATATGCCGAGCACATCAGGTGGTAGAGGATGGATACGAGTTCTTTGCGGAAAGGAGATTAGTCACGATCTTCTCAGCTCCAAACTACTGTGGAGAATTTGATAATGCGGGTGCTCTGTTGAGCATAGATGAGAGCTTAATGTGTTCTTTCCAGATCTTGAAGCCAAAAGAAACAGGCGCACCACATTCAAGAAAACCAAATACAAACAAGGCACCGAGAGCGGAAGATGGTTAACTTCGTATCCAGTCACATAGTCATAACTGTTGTCAGCTCGAAGGGGGTGATCTCCTATATCTCAGATCCACCAGGGCATGGCATTAGAAGCACTCTCCAGCCCAGTTCTCTTACCCTCTCCGCTACTTGCTGGCGGTACTGCACTCTTCTGTATAGCAGTGTCTGGCTCGCATTCTTGTTGTGGATGCAGTAATTTCGTTGTTGTTAGTTTGTTAGTGTTGTTTCTTTTGCCTGATTGCTGTGAGCTATGTTTCCTTGACAATGTATGGTTGGTGGAGTTGTATGCAGTTGACCCTGGCACCTGTGAAAATTGAACGGGGTACGTTTTTGGTGTGTAGTTTGGTGATCCCTCGGCTTGGAGCTGCACAAGCTACTTAGTACATGCTCCCTACAATTCACAAAACACATTATATTTTTTTTCTCATTTATTTCTTAATATAGCTCATTTCTCT

>Bradi2g35150 Org_Bdistachyon transcript: Bradi2g35150.2.p NF-kappaB-inducing kinase activity, myosin phosphatase activity (Blast2GO) (PAC:32775198)

CGGCAAATCAAAGACGGAGATGTGTTCACGCGGCTTTGAACGATCAAAACGATTGAAGTATCAATTTTGTCCTCGTCAGAAACACAACTATTCCCGACAAGCCCCCGTTTACCAATCCCCTGCCCCCCCTTCCCCCTTCGTCCCCCCTGCGGCCGGGAAGATGATGATGACGCGGGCATCGATGGGGGCCATGGAGGGATCCGCGGTGGACGAGGTGGTACGGCGGCTGGTGGAGGGCGGTCGCAGCGGGCGGCAGGTTCAGCTGTCGGAGGCGGAAATCCGGCAGCTCTGCGTCGACGCCAAGCGGGTGCTCCTCTCCCAGCCCAACCTCCTCCGCATCCACGCCCCCGTCAAGATCTGCGGTGATATCCATGGTCAGTTTGTTGATCTGTTGAGGTTGTTTGATTTGGGTGGTTATCCTCCAACTTCCACTTACTTATTCCTCGGAGACTACGTAGATAGAGGCAAACAGAGTTTGGAAACTATATGCTTGCTGCTGGCATACAAAATAAGGTACCCTGACAAGGTTTTCCTGTTAAGGGGGAACCACGAAGATGCAAAAATCAACAGAGTTTATGGTTTCTATGATGAGTGCAAGAGGAGGTTCAACGTACGACTGTGGAAGATATTCTCTGATTGCTTCAACTGCCTGCCTATTGCAGCACTCATTGATGATAAGATACTATGCATGCATGGTGGCCTGTCACCTGAACTGAATGATCTGGACCAAATAAAGGATATTGAGAGACCAGCTGAGATTCCTGATTATGGTCTCCTATGTGATTTGCTTTGGTCTGATCCTAGTCCCCATGGAGAAGGGTGGGGGGAGAGTGACAGAGGTGTTTCGTGTACGTTTGGTGCGGATAAGCTTGTAGAGTTTTTGGAGAAGAATGATCTTGACCTTATTTGCCGAGCACATCAGGTGGTAGAAGATGGTTACGAGTTCTTTGCACAGCGGAGATTAGTGACAATCTTCTCAGCCCCAAATTATTGTGGGGAATTCGATAATGTAGGTGCTCTGTTGAGCATAGATGAAAACCTAATGTGTTCATTTCAAATCTTGAAGCCAACGGAAACAGGCATACCACGTGTAAGGAGATCAATTCCAAATAAGCCAGCATGTGGGGAAAACTCCTGATCAACATCCAATTCCCTCAATATTATTTTCGTCGGCTCAAAGGGGGCAATCTGATCTATTGTTTAGATCCACCAGCCAATCATCTTGCCCTAATTTCTCTTAATTCCTAAGCATTTGTTTGCTCTGCACTACAAGTTCTTCTGTACAGCATGTTTGTGAGTTGATGTAGCTTTTAACATTTTATATACCATTATTTCTTATTTTCTTTCAGTTTTGGTTCAGGCTACTAATCTATTATTTTAATGCAGTGTAAAGGAAAAGTTTGTTTTTTGTTTCTTTTTATCTGCCATATATTTGTGTCAAAAGAAAGATGATTTTTGGCATTCTGCAACGGAAGCAACCAAAC

>Bradi3g37570 Org_Bdistachyon transcript: Bradi3g37570.1.p NF-kappaB-inducing kinase activity, myosin phosphatase activity (Blast2GO) (PAC:32820716)

AGGGAGCCTAGGAGCCAAGCAATTGCCGGGCATAAAACGAAAGTCATGGACATGGACGCACGAACTTTCCTCTCCTCGACCCCCCTCCTCTTCTCCTTCCCCGTGCGCCCTCCTCCTCCTCTGCTCCCTTACCGCGCGTCGCCGGCGTCGATCCCCCTCCGCACCGCGCGCGGCAAGGGAGACAGGGAGAGCGCGTGACTCATGGACGGGAACGCCCTCGAGGAGCTGATACGGCGGCTCCTGGACGGGAAGAAGAACAAGGGGCCGGGGAAGAAGGTGCAGATCAGCGAGGCGGAGATCCGGCACCTCTGCGTCACCGCCAAGGAGATCTTCCTCTCCCAGCCCAACCTCCTCGAGCTCGAGGCCCCCATCAACGTCTGCGGCGACATCCACGGGCAATTCTCGGACCTGCTGCGGCTGTTCGACTACGGCGGGCTGCCGCCGAGCGCCAACTACCTGTTCCTGGGCGACTACGTGGACCGGGGCAAGCAGAGCATCGAGACCATCTGCCTGCTGCTGGCCTACAAGATCAAGTTCCCGGACAACTTCTTCCTGCTGAGAGGGAACCACGAGTGCGCCTCCATCAACCGAATCTACGGCTTCTACGACGAGTGCAAGCGCCGCTTCAGCGTGCGCCTCTGGAAGCTCTTCACCGACTGCTTCAACTGCCTCCCCGTCGCCGCCGTCATCGACGACAAGATCCTCTGCATGCACGGCGGCCTCTCCCCGGACCTCGACAGCCTCGACCGGATCAGGGAGATCCAGCGGCCCGTCGACGTCCCCGACCAGGGCCTCCTCTGCGACCTCCTCTGGTCCGACCCCGACCGCGAGAGCTCCGGATGGGGCGAGAACGACCGCGGCGTCTCCTTCACCTTCGGCGCCGACAAGGTCGCCGAGTTCTTGAACAAGCACGACCTCGACCTCATCTGCCGCGCTCACCAGGTTGTGGAGGACGGGTACGAGTTCTTCGCGGACCGGCAGCTGGTGACAATATTCTCCGCGCCCAACTACTGCGGGGAGTTCAACAACGCCGGCGCGCTCATGAACGTCGACGCCAGCCTGCTCTGCTCCTTCCAGATCCTCAAACCGTTGAGAGGCAGATCGCAGACGGAGGGATGAAAGGAGGGCCGCCGATCCAGGCAGAGATTTTGATTGTTAGAGGCTTTGCTTAGAATCATGGAGATCTCGTAGATTTGCCTGCACAGGCTTCTCCGATCCGCCCAGTTTTTGTTGTTTGGGGGAACTGTTGATCACTTCGTACATCCTGTTCTTCTGAATCCTTTGCTTGTAACTGATTCTATACGTGTATATAGAAGCATATAGTTTCTTAATTGCTCTGCGTGTAAATTCAGGATTCAAGCCAGATGCAAGTTGCCATCTGAAACTGCAGGTAAAC

>Bradi3g55614 Org_Bdistachyon transcript: Bradi3g55614.1.p (M=4) PTHR11668:SF280 - SERINE/THREONINE-PROTEIN PHOSPHATASE PP1 ISOZYME 7 (PAC:32814550)

GGGGAATCGGCACAGGAGAGAGAGAAAGGGAGAGTTAGAGAGAGAAGGGAGGGGGAGAGGAGAGGGAGACCCAATATCCATGCAAAGAACAAGAAATCAGAGAGGCAAGGCAAAATTCCAGTCCAAATCCCCTCCAAAAACCCACCAAACCAGCTCCCCCATCTCCAAGAACAAGAACAAGAAAGCATCCTTGAGCACAAGGGAATAAAGGAGCCCGGAAAGGCGAAGACAAAAGGTACTATCTTTGAACCATTTCAATATACTGATCGCAAGCCAGGAGCCGCCTCCTCCCCAAATCCCTCAAAAGAGCAAAGCAAACAAGATTTATCCGATCTGTGCTTTCAGTTTTTTGACCGCAAAGTTCTCGTTTCCTTTTCTGTAACCGTTGCTGCTCTTGCGTTCTTGAGAAGTTTCAAGGAGAGCTCGGAGAAGGGGATGGATCCGGCGCTGCTCGACAACATCATTGGGAGGCTGCTCGAGGTAAAGAGCCTGAAGCCCGGGAAGAACGCGCAGCTGTCGGAGTCGGAGATTAAGCACCTATGCGCCGCCTCCAAGGAGATCTTCCTCTCGCAGCCCAACCTCCTGGAGCTCGAGGCCCCCATCAAAATCTGCGGTGATGTCCATGGTCAATATTCTGATCTCCTGAGGCTCTTTGATTATGGTGGATATCCTCCTGAATCCAACTATCTTTTCTTGGGGGATTATGTGGACCGGGGAAAACAAAGCCTTGAGACAATATGCCTTCTTTTGGCTTATAAAGTCAAGTACCCTGAGAACTTTTTTCTTCTAAGAGGCAATCATGAATGTGCATCAGTAAACCGCATCTATGGGTTTTATGATGAGTGCAAGCGCAGATTCAGTGTAAAACTCTGGAAAACATTTACAGACTGTTTTAACTGCTTGCCAGTATCAGCATTGATAGATGAAAAGATTCTATGCATGCATGGTGGTCTTTCTCCTGAATTGAACAAGCTGGATCAAATACTCAACCTCAATCGCCCCACAGATGTACCAGATACTGGATTACTGTGTGACCTTCTTTGGTCAGATCCTTCCAATGAAGCGCAAGGGTGGGCTATGAATGATCGAGGTGTTTCATATACATTTGGTCCTGATAAAGTGGCTGAGTTTCTTGAGAAGCATGATTTAGACCTCATCTGCCGAGCTCATCAGGTTGTTGAAGATGGATATGAGTTCTTTGCTGATCGTCAGCTTGTAACAATATTTTCTGCGCCTAATTACTGTGGAGAATTCGATAATGCTGGTGCCATGATGAGTGTTGATGAGACGCTAATGTGCTCCTTCCAAATACTGAAACCTGCGAGGAAAGTGTTGAGTGGTTCAACTAATACCAAGTCTGGCTTCAAGTCATTGAGAGGCTGGTGATGGCGGTACGGTGCTTGGTGAAAGGTGAGCAAGCTGTGACCAGATCTGCAGGTAGGCACATACCTTGAGGAGCGGCTGCAACTAACTGGCATTTTCGCCTACACCTCAGGGCCCGAAACAAGAACCACAGAAATGTACAACTCTCGTCGATGGAACCGTACATTGTTCGTGTCGGATTAACACTCTTGTAATGTATTATTGTTTTTCTGCCGTGTGGATCTCTTATCGCGCAAGAGGATATGCTGTGAACTCTTACTGTTCTTCCCCCTCTCCCCTTTCCCCTATAAAAAATTCTAAGGATTCAGGATTCCAACAGGCGTACGGCAGTAAAACGGCTGGGAATCCTGAGTACTCTTTGGACCCAAATTTTCATCCCAGCGAACAAGGGCGTTGCAGCCTTGCTGGGCACAATATCATAGCAGGCGGCAACACT

>Traes_1AS_4025A300E Org_Taestivumearly-release transcript: Traes_1AS_4025A300E.1 (M=6) PTHR11668:SF262 - SERINE/THREONINE-PROTEIN PHOSPHATASE PP1 ISOZYME 8-RELATED (PAC:31959549)

CGATTAATTAAGCTCGTGGTCGTCACAAACGCAACTATTCCCAGGCCATCCCATTTCCAATCCCCCCACCCTTCCTCTCTCTCCCCCGCCCCTCGCCGTCGCCGGAGAGAGAGAGAGGGACGCGGCCGCGGGAGAAGAAGGAGGGAAGATGATGATGACACGGGCCTCCATGGGGGCCATGGAGGGCGCGGCGCTGGACGAGGTGGTGCGGCGATTGGTCGAGGGCGGCCGCGGCGGGCGGCAGGTGCAGCTGTCCGAGGCGGAGATCCGGCAGCTCTGCGTCGACGCCAAGCGGGTTTTCCTGTCCCAGCCCAACCTCCTCCGCATCCAGGCCCCCGTCAAGATCTGCGGTGATATCCATGGTCAGTTTGTTGATCTTCTAAGGTTGTTTGATTTGGGTGGTTATCCTCCAACCTCAACCTATGTATTCCTCGGAGACTACGTAGATAGAGGCAAACAGAGCTTGGAAACTATATGCTTACTGCTGGCATACAAAATAAGGTACCCTGACAAGGTTTTCCTGTTAAGGGGGAACCATGAAGATGCAAAAATCAACAGAGTTTATGGTTTCTATGATGAATGCAAGAGGAGGTTCAATGTTCGACTGTGGAAGATATTCTCTGATTGCTTCAACTGCCTGCCTATTGCAGCACTCATTGACGACAAGATACTGTGCATGCATGGTGGCCTTTCACCTGAACTGACTAACCTGGACCAAATAAAGGATATTGAGAGGCCGGCTGAGATTCCTGATTATGGTCTCCTGTGTGATTTGCTTTGGTCTGATCCTAGCCCTGACGGAGAAGGGTGGGGTGAGAGTGACAGAGGTGTTTCATGTACGTTTGGTGCAGATATGCTTATAGAGTTTTTGGAAAAAAATGATCTTGATCTTATCTGCCGAGCCCATCAGGTGGTAGAAGATGGTTACGAGTTCTTTGCACAGCGGAGATTAGTCACAATCTTCTCAGCTCCAAATTATTGTGGGGAATTCGATAATGTAGGTGCTCTGTTGAGCATAGATGAAAATCTAATGTGCTCATTTCAAATCTTGAAGCCAAATGAAACAGGCACACCGCGTGTGAAAAGACAAATCCCAAATAAGCCAGCAATTGGGGGAAGTGCCTGATCTGATAAACATCCAATTCCTTCAATATTACCATTGTCAGCTCGAAGGGGGTGATCTGATCTATTGTTTGGATCCACCAGGGCATGGCAATATTCATCCACCAGCCAATTGTCTTGCTTAACCACCCTTAGTTGCCGAGCATTTGTAAATGTCCATTGGCTCTG

>Traes_1BS_BF71914E7 Org_Taestivumearly-release transcript: Traes_1BS_BF71914E7.1 (M=6) PTHR11668:SF262 - SERINE/THREONINE-PROTEIN PHOSPHATASE PP1 ISOZYME 8-RELATED (PAC:31771990)

GTGGTCGTCACAAAACGCAACTATTCCCAGGCACATCCCATTTCCAATCCCCCCACACTTCCTCTCTCTCTCCCCCGCCCCTCGCCGTCGCCGGAGAGAGAGAGAGAGGGACGCGGCCGCGGGAGAAGAAGGAGGGAAGATGATGATGACACGGGCCTCCATGGGGGCCATGGAGGGCGCGGCGCTGGACGAGGTGGTGCGGCGATTGGTCGAGGGCGGCCGCGGCGGGCGGCAGGTGCAGCTGTCCGAGGCGGAGATCCGGCAGCTCTGCGTCGACGCCAAGCGGGTTTTCCTGTCCCAGCCGAACCTCCTCCGCATCCAGGCCCCCGTCAAGATCTGCGGTGATATCCATGGTCAGTTTGTTGATCTTCTGAGGTTGTTTGATTTGGGTGGTTATCCTCCAACCTCAACCTATGTATTCCTTGGAGACTACGTAGATAGAGGCAAACAGAGCTTGGAAACTATATGCTTACTGCTGGCGTACAAAATAAGGTACCCTGACAAGGTTTTCCTGTTAAGGGGTAACCATGAAGATGCCAAAATCAACAGAGTTTATGGTTTCTATGATGAATGCAAGAGGAGGTTCAATGTTCGACTGTGGAAGATATTCTCTGATTGCTTCAACTGCCTGCCTATTGCAGCACTCATTGATGACAAGATACTGTGCATGCATGGTGGCCTTTCACCTGAACTGACTAACCTGGACCAAATAAAGGATATTGAGAGGCCGGCTGAGATTCCTGATTATGGTCTCCTGTGTGATTTGCTTTGGTCTGATCCTAGCCCTGACGGAGAAGGGTGGGGGGAGAGTGACAGAGGTGTTTCATGTACGTTTGGTGCAGATAAGCTTATAGAGTTCTTGGAAAAAAATGATCTTGATCTTATCTGCCGAGCCCATCAGGTGGTAGAAGATGGTTACGAGTTCTTCGCACAGCGGAGATTAGTCACCATCTTCTCAGCTCCAAATTACTGCGGGGAATTCGATAATGTAGGTGCTCTGTTGAGCATAGATGAAGATCTAATGTGCTCATTTCAAATCTTGAAGCCAAATGAAACAGGCACATCGCGGGTAAAAAGACAAATTCCAAATAAGCCAGCAACCGGGGGAAGTGCCTGATCTGATCAACATCCATTCCTTGAATATTAGCATCGTCAGCTCGAAGGGGGTGATCTGATCTATTGTTTGGATCCACCAGGGCATGGCAATATTCATCCACCAGCCAATTGTCTTGCTTAACCACCCTTAGTTGCCGAGCATTTGTAAATGTCCATTGGCTCTGCACTCGTTTTCTCTGTACAGAATTAGGCCGGATTTAACAGCACCACCTATTCTGGTTGTTCTTCCTGGTTGCGCGGCATCTGTTTTTGTAAGTTACTTTTGTGGTACATTTTTGCCAGTGGATGTTGATAGCTTCTGTTTGTGAGCATCTATTATTTGCCTTATGTTTGTGACGTGACGTTTTACCAACTTGTTCTACACTTCTACTTACAATCTTGTGGTCTGTGAGGGAAGGTCTCCAAGGTTCTTATGACCTTGTTTGTTTTTTCTTTCATTTTTTGCATGTTCG

>Traes_1DS_309F807A1 Org_Taestivumearly-release transcript: Traes_1DS_309F807A1.2 (M=6) PTHR11668:SF262 - SERINE/THREONINE-PROTEIN PHOSPHATASE PP1 ISOZYME 8-RELATED (PAC:31868116)

ATTAAGCTCGTGGTCGTCACAAACGCAAACTATTCCCAGGCACATCCCATTTCCAATCCCCCACCCTTCCTCTCTCTCTCCCCCGCCCCTAGCCGTCGCCGGAGAGAGAGAGGGACGCGGCCGCGGGAGAAGAAGGAGGGAAGATGATGATGACACGGGCCTCCATGGGGGCCATGGAGGGCGCGGCGCTGGACGAGGTGGTGCGGCGATTGGTCGAGGGCGGCCGCGGCGGGCGGCAGGTGCAGCTGTCCGAGGCGGAGATCCGGCAGCTCTGCGTCGACGCCAAGCGGGTTTTCCTGTCCCAGCCGAACCTCCTCCGCATCCAGGCCCCCGTCAAGATCTGCGGTGATATCCATGGTCAGTTTGTTGATCTTCTGAGGTTGTTTGATTTGGGTGGTTATCCTCCAACCTCAACCTATGTATTCCTCGGAGACTACGTAGATAGAGGCAAACAGAGCTTGGAAACTATATGCTTACTGCTGGCCTACAAAATAAGGTACCCTGACAAGGTTTTCCTGTTAAGGGGTAACCATGAAGATGCAAAAATCAACAGAGTTTATGGTTTCTATGATGAATGCAAGAGGAGGTTCAATGTTCGACTGTGGAAGATATTCTCTGATTGCTTCAACTGCCTGCCTATTGCAGCACTCATTGACGACAAGATACTGTGCATGCATGGTGGCCTTTCACCTGAACTGACTAACCTGGACCAAATAAAGGATATTGAGAGGCCGGCTGAGATTCCTGATTATGGTCTCCTGTGTGATTTGCTTTGGTCTGATCCTAGCCCTGACGGAGAAGGGTGGGGGGAGAGTGACAGAGGTGTTTCATGTACGTTTGGTGCAGATATGCTTATAGAGTTCTTGGAGAAAAATGATCTTGATCTTATCTGCCGAGCCCATCAGGTGGTAGAAGATGGTTATGAGTTCTTCGCACAGCGGAGATTAGTCACCATCTTCTCAGCTCCAAATTACTGCGGGGAATTCGATAATGTAGGTGCTCTGTTGAGCATAGATGAAAATCTAATGTGCTCATTTCAAATCTTGAAGCCAAATGAAACAGGCACATCGCGGGTAAAAAGACAAATTCCAAATAAGGTAAATCAGCTGGCTTCAGATTGGATTCATATTGTACTATGCACTAGAAGTTGAACCTGTGGAGATAGAATGTTTTATCATTGTATTATCTTTATGGACTACTCCCTCTGTGCATAAATATAAGACGTTTTAGAGACTTGTCAAGTCTCTAAAATGTCTTACATTTATGAACAGAGGGAGTAGAATATTAGAAAAATAGAAACCATGATCAATGAAACGAAAGAAAATTCGATGATAATGCCTCTGATGCACAAGATCTGCTGTGGTTTGCTGGTTCTGTTAATTATTGCATAATCCAGTCATCTGCCCTTTTTTAATGAACATACAATCGCTGCACAGTGGGCCGCACATTAATTTTTTTTCTAAATTTTAATGCCATCTCGATGTTACTTCTGTATGTTCACGAGGAAACTTTGCGGATGACACTACCACAGAGAGTGATAGTGAATTGATAATGTAATTGAGGATAATCCTTCATTTAATCTTTGATCAAGAAGGGGCTATTCAATGCATTCATTGTATTTCGGCCAACTATTACATTTTAGCCGTTAGGCCAACTCCAACGCAGTGACCCAAACGGACACGGATTTTATCCGTATTTTGTCAGTTTGGGTCAGGAAAACGGACATCGGGTGCGAGCGGACAGACGGACACGACCTCTGCGCCC

>Traes_3AS_8B6A13B23 Org_Taestivumearly-release transcript: Traes_3AS_8B6A13B23.1 (M=6) PTHR11668:SF262 - SERINE/THREONINE-PROTEIN PHOSPHATASE PP1 ISOZYME 8-RELATED (PAC:31888541)

AAAAAATACTACTAGTAGTAGATAAACAACCTAATGACCACACAAAAAAACCATATTTACCACGTTCGTTAAAAAAAAACCATAATTACCACGCCATTATCCAATCCCGACCACCTCGTCGTCCATTTTTTTTTTCCGAGAGTACCCTCCTCCATTTCTAATCCTCCTCGCACCTCCGTCGTCGTCTAGGGATCGAGCCGGTGGAGAGCGGTGAAGATGATGATGACGCGGGCATCGATGGGCGCCATGGATGGGGCCGCGTTGGATGAGGTGGTTCGGCGGCTCGTCGAAGGGGGCCGCGGCGGGCGCCAGGTCCAGCTGTCGGAGGCGGAGATCCGCCAGCTCTGTGTCGAGGCCAAAAGGGTGCTCCTCTCGCAGCCCAACCTCCTGCGCATTCCCGCGCCCGTCAAGATCTGCGGTGATATCCATGGTCAGTTTGTTGATCTTCTGAGGCTGTTCGATTTGGGTGGCTATCCTCCAACTTCGACTTATCTTTTCCTTGGAGACTACGTGGATAGAGGCAAACAAAGCTTGGAAACCATATGTCTGCTTCTGGCGTATAAAGTGAAGTACCCTGATAAGGTTTTCCTGTTGAGAGGAAACCATGAAGATGCAAAAATTAACAGAGTTTATGGTTTCTATGATGAATGCAAGAGGAGATTCAATGTTCGTCTGTGGAAGATATTCTGTGATTGCTTCAACTGCTTGCCTATGGCAGCGCTTATTGATGATAAGATATTCTGTATGCATGGTGGCCTCTCACCTGAATTGAATAGCTTAGATCAAATTAAGGATATTGAGAGGCCTGTTGAAATTCCTGACTATGGTCTTCTATGTGATTTGCTTTGGTCTGATCCTAGTTCTGACACACAAGGGTGGGGGGAGAGTGACAGAGGTGTTGCTTGTACTTTCGGTGCGGATAAGCTTGTAGAATTTTTGGAGAAGAATGATCTTGACCTCATTTGCCGAGCTCACCAGGTGGTAGAGGATGACTATGAGTTCTTTGCAGAAAGGAGATTAGTCACCATCTTTTCAGCTCCAAACTACTGTGGAGAATTTGATAATGCGGGTGCTTTGTTAAGCATAGATGAAAGCTTAATGTGTTCTTTCCAGATCTTGAAGCCAAAAGAAACAGGAGCACCACATTCAAGAAAACCAATTTCAAACAAGG

>Traes_3B_9B97F74FC Org_Taestivumearly-release transcript: Traes_3B_9B97F74FC.2 (M=6) PTHR11668:SF262 - SERINE/THREONINE-PROTEIN PHOSPHATASE PP1 ISOZYME 8-RELATED (PAC:31892846)

GTCCTACATTATTGATCTTACGCGGAGATTTGTGTGAGAATTTTCTTTTCTATGTTTGTTTTTTTGCTTGATTAAGTCGCTTAGACATGCTGGCGTAGAAAAAAAATATAAACAACCTAATTACCACACCCTAAAAAAACCATTTTTACCACGTTCGTTAAAAAAACCATACCAATCCCGACCACCTCCTCCTCCATTTTTTTTTCCGAGAGTACCCTCCTCCATTTCTAATCCTCCTCGCACTTCCGTCGTCGTCTAGGGATCGAGCCGGCGGAGAGCGGTGAGGATGATGATGACGCGGGCATCGATGGGCGCCATGGATGGGGCCGCGTTGGATGAGGTGGTTCGGCGGCTCGTCGAAGGGGGCCGCGGCGGGCGCCAGGTCCAGCTGTCGGAGGCGGAGATCCGCCAGCTCTGCGTCGAGGCCAAAAGGGTGCTCCTCTCGCAGCCCAACCTCCTGCGCATTCCCGCGCCCGTCAAGATCTGCGGTGATATCCATGGTCAGTTTGTTGATCTTCTGAGGCTGTTCGATTTGGGTGGCTACCCTCCAACTTCGACTTATCTTTTCCTTGGAGACTACGTGGATAGAGGCAAACAAAGCTTGGAAACCATATGTCTGCTTCTGGCGTATAAAGTGAAGTACCCTGATAAGGTTTTCCTGTTGAGAGGAAACCATGAAGATGCAAAAATTAACAGAGTTTATGGTTTCTATGATGAATGCAAGAGGAGATTCAATGTTCGTCTGTGGAAGATATTCTGTGATTGCTTCAATTGCTTGCCTATGGCAGCGCTTATTGATGATAAGATATTCTGTATGCATGGTGGCCTCTCACCTGAATTGAATAGCTTAGATCAAATTAAGGATATTGAGAGGCCTGTTGAAATTCCTGACTATGGTCTTCTATGTGATTTGCTTTGGTCTGATCCTAGTTCTGACACACAAGGGTGGGGGGAGAGTGACAGAGGTGTTGCTTGTACTTTCGGTGCGGATAAGCTTGTAGAATTTTTGGAGAAGAATGATCTTGACCTCATTTGCCGAGCTCATCAGGTGGTAGAGGATGGCTATGAGTTCTTTGCAGAAAGGAGATTAGTCACCATCTTTTCAGCTCCAAACTACTGTGGAGAATTCGATAATGCGGGTGCTTTGTTAAGCATAGATGAAAGCTTAATGTGTTCTTTCCAGATCTTGAAGCCAAAAGAAACAGGAGCACCACATTCAAGAAAACCAATTTCAAACAAGGTAAATAATCAGGCTTCAGTTGGTTTCCCTTTCATGACCCAATATATTGGACAAGTGACAGATAGGCCATCAGTGAGTTGTTCTTGTTTGAACTACAATTCATGAAAAA

>Traes_4AL_2EBB63DBA Org_Taestivumearly-release transcript: Traes_4AL_2EBB63DBA.1 (M=10) PTHR11668:SF280 - SERINE/THREONINE-PROTEIN PHOSPHATASE PP1 ISOZYME 7 (PAC:31881030)

CTTTGGCTGGCCTGCTTTGGGTGGTCGCTGCCTCTCTGCTAATATTCCTTGGAACTAGCTAGCTTGCAACATACTCAGAGCAGGACAGAGCATAGGGTGCTCCATAACCCATACCAGCCAGCAGAAAAGATTAATTATCAAGCCTCATGGTCGTCGTCGTCGTCGGCGGCGGCTTATAAACATCCATTGCTGCTGGACCGTCAGACCTTCAAGTAGGAGCTAGCTCCAGTTGGTTTGCTTGCTTGATTGGCGATGAACGGTGCGGCGCTAGACAACGTGATCCGGCGGCTGCTGGAGGTGCGGCGGGGGCGGCCGGGGAAGCAGCAAGTGCAGCAGGTGCAGCTGGGCGAGGGGGAGATCCGGCAGCTCTGCGGCGCCGCCAAGGACGTCTTCATGCGCCAGCCCAACCTGCTCCAGCTCGACGCCCCCATCAAGATCGCCGGTGACATCCACGGGCAGTACACGGATCTCATCCGACTCTTCGAGCTGGGCGGCTTCCCGCCGCAGCACAAGTACCTGTTCCTGGGCGACTACGTGGACCGCGGCAAGCAGAGCATCGAGACCATCTGCCTGCTGCTCGCCTACAAGCTCCGGTACCCGGAGCACTTCTTCCTCCTCCGCGGCAACCACGAGTGCGCCTCCGTCAACCGCGTCTACGGCTTCTACGACGAGTGCAAGCGCCGCTACTCCGTCCGCCTCTGGCGCCACTTCTCCGACTGCTTCAACTGCATGCCCGTCGCCGCCCTCGTCGAGTCGCGCATCCTCTGCATGCACGGCGGCCTCTCCCCGGACCTCCGCCACATCCGCGACATCGCCGGCCTCCCCAGGCCAGTCGACGTCCCCGACACCGGCCTCCTCTGCGACCTCCTCTGGTCCGACCCCGGCGGCGCCGCGGGGTGGGGACCCAATGAGAGGGGCGTGTCGTACACGTTCGGGGCGGACGTGGTGGCGGCCTTCATGGAGAGGCACGACCTGGACCTCGTGTGCCGGGCGCACCAGGTGGTGGAGGACGGCTACGAGTTCTTCGCCGGGAGGAGGATGGTCACCGTCTTCTCCGCGCCCAACTACTGCGGCGAGTTCGACAACGCCGGCGCCCTCATGTGCGTCGACGACGACCTCACCTGCTCGTTCCAGATACTCAAGCCCGCGGACAACAGGCAGCGGCGTTTCGCCTTCGGCATGGGATCATCCGCATCTACTACTAGCAGGGGGATCCGATCACCGTGGTGCTAATAATTAATATGCATGCTTTGCTTGGTCAAAACGGATGCATGCAATAAACCCATCCATCTTTGATTTCGATCCTTGGCAATTTTGTATACCACAGTTTTTTGTATACCACAGTAAGAGTTTTCTTGCATGTATTCGCAAAAAAAAAAGAGTTTTCTTGCATGCATGCATGGTGTAGCTCACATAAACAACAGGAAAAGCAAGGCTTGTATTTCATTTGGAGGCATCCGGACACTCTTTTTGTAGGGTTATATAAACTAATGTCATGCAAATTTAGTTTTGTT

>Traes_4AS_6D7CDA716 Org_Taestivumearly-release transcript: Traes_4AS_6D7CDA716.1 (M=6) PTHR11668:SF259 - SERINE/THREONINE-PROTEIN PHOSPHATASE PP1 ISOZYME 2-RELATED (PAC:32023484)

CCATCGGCGGCCCACCACTCAATCTCTCTCCTCCCCCTCCTCGGCCACCTGCTCCTCCCGAAGCGCCGAAACCCTAACTCCACCGATCTCTCTCCACCCCGGGGGAGGCGATGGCGGCGGCGCCGGCGGCGGGAGGGCAGGGAGGCGGCATGGACGCCGCGCTCCTCGACGACATCATCCGCCGTCTGCTCGAGGTGCGGACGGCGCGCCCCGGCAAGCAGGTGCAGCTCTCCGAGTCGGAGATCCGCCAACTCTGCACCGCCTCCCGCGACATCTTCCTCACCCAGCCCAACCTCCTCGAGCTCGAGGCGCCCATTAAAATCTGCGGTGATATCCATGGTCAGTACAGTGATCTTTTAAGGCTATTTGAGTATGGAGGTTTTCCCCCAGAAGCCAACTATCTATTCTTAGGCGATTATGTTGATCGAGGCAAACAGAGTCTGGAGACTATATGCCTCCTCCTTGCATACAAAATCAAGTACCCCGAGAACTTTTTTCTTCTGAGAGGCAACCATGAGTGTGCTTCAATAAACAGAATATATGGATTTTATGATGAATGCAAGCGTCGCTTCAATGTGCGGCTATGGAAGGTCTTCACCGACTGTTTTAATTGTCTCCCTGTGGCCGCTCTAATCGATGATAAAATATTATGCATGCATGGTGGCCTTTCTCCTGATCTGGGACACCTAGATGAGATAAAAAACTTGCCCCGTCCTACCGATGTGCCAGATACAGGTCTACTATGCGATCTTCTTTGGTCTGATCCAGGAAAAGATGTCCAAGGGTGGGGCATGAATGATAGGGGCGTTTCATACACATTTGGCCATGACAAAGTTACGGAGTTCCTTCTAAAGCATGATCTTGATCTTATTTGCCGTGCCCACCAGGTTGTCGAGGATGGGTATGAATTCTTTGCTGACAGGCAACTGGTCACCATATTTTCGGCTCCCAACTATTGTGGTGAATTTGATAATGCTGGAGCAATGATGAGTGTTGATGAAACTTTGATGTGTTCATTTCAAATTCTCAAACCTGCTGAGAGAAAAATCAAATTTATGGCGTCAAACAAAATGTGAAATGATGAAGCTCTTTCCTCTTCTAGAGGCTCTGAGGCTCTCATGTGGACACAATCACATCAAGACATCAACGCGCAGGCGCTATGCTTGAAGGCAGCATCGGGCTAGTTCTACTGGATTAGTCAAATAATATCATTTCTAACAGTTGGTCTGAAAGTTACTCTTCATAGCAAGTGCCAGAATTTTTTTCCTTTTCTGTTCCCACCGGGTGTGAAACTATCTTGTAGCTGTAGATATATTGATTATGGAAGGCTGAAGTTCGCAATTGATGCAAGATGAT

>Traes_4BL_3AA55AD10 Org_Taestivumearly-release transcript: Traes_4BL_3AA55AD10.1 (M=6) PTHR11668:SF259 - SERINE/THREONINE-PROTEIN PHOSPHATASE PP1 ISOZYME 2-RELATED (PAC:31913024)

ACCCAATCTCTCTCCTCCCCCTCCTCGGCCACCTGCTCCTCCCGAAGCGCCGAAACCCTAACTCCACCGATCTCTCTCCACCCCGGGGGAGGCGATGGCGGCGGCGCCGGNGGGAGGGCAGGGAGGCGGCATGGACGCCGCGCTCCTCGACGACATCATCCGCCGTCTGCTCGAGGTGCGGACGGCGCGCCCCGGCAAGCAGGTGCAGCTCTCCGAGTCGGAGATCCGCCAGCTCTGCACCGCCTCCCGCGACATCTTCCTCACCCAGCCCAACCTCCTCGAGCTCGAGGCGCCCATCAAGATCTGCGGTGATATCCATGGTCAGTACAGTGATCTTTTAAGGCTATTTGAGTATGGAGGTTTTCCCCCGGAAGCCAACTATCTATTCTTAGGCGATTATGTTGATCGAGGCAAACAGAGTCTGGAGACTATATGCCTCCTCCTTGCATACAAAATCAAGTACCCTGAGAACTTTTTTCTCCTGAGAGGCAACCATGAGTGTGCTTCAATAAACAGAATATATGGATTTTATGATGAATGCAAGCGTCGCTTCAATGTGCGGCTATGGAAGGTCTTCACCGACTGTTTTAACTGTCTCCCTGTGGCCGCTCTAATTGATGATAAAATATTATGCATGCATGGTGGCCTTTCTCCTGATCTGGGACACCTAGATGAGATAAAAAACTTGCCCCGTCCTACCGATGTGCCAGATACAGGTCTACTATGCGATCTTCTTTGGTCTGATCCAGGAAAAGATGTCCAAGGGTGGGGCATGAATGATAGGGGCGTTTCATTCACATTTGGTCATGACAAAGTTACGGAGTTCCTTCTAAAGCATGATCTTGATCTTATTTGCCGTGCCCACCAGGTTGTCGAGGATGGGTATGAATTCTTTGCTGACAGACAGCTGGTCACCATATTTTCGGCTCCCAACTATTGTGGTGAATTTGATAATGCTGGAGCAATGATGAGTGTTGATGAAACTTTGATGTGTTCATTTCAAATTCTCAAACCTGCCGAGAGAAAAATCAAATTTATGGCGTCAAACAAAATGTGAAATGATGAAGCTCTTTCCTCTTCTAGAGGCTCTGAGGCTCTCATGTGGACACAATCACATCAAGACATCAACGCGCAGGCGCTATGCTTGAAGGCAGCATCGGGCTAGTTCTACTGGAGAAGTCAAATAATATCATTTCTAACAGTTGGTCTGAAAGTTACTCTTCATAGCAAGTGCCAGAATTTTTTCCTTTTCTGTTCCCACCGGGTGTGAAACTATCTTGTAGCTGTAGATATATTGATTATGGAAGGCTGAAGTTCGCAATTGACGCAAGATGATCCCCTCCAGCAAGAAACAAAAAGTTCTGAATATGAGAGTTTTCTGTTTGGGAGTTCCACTATCTAAAAAGCACCTGTATCACTTACTGAGGTAAGAACAAAAGCCAAGCCTGTGTTTCTGTTGCTGTAAGCAAATATTGGATGAGTTTTATTTTAGTTTTTAGAGAGCGGTAATGGTAACAATTCAAATAGTATGAGCACAAAGAAAGCTGATGCTTGGTGGGAACTTGATGAGAAAATATTCAGCATGTTCCTGATGTAGGCTTCACCTTTCAAGTGCTGGATGGCTTTGAGTTTTTGTTCAATCAGACCAGCATGATGGGCTGTTGCTTGATTAGCATTAACGTGGAAACA

>Traes_4DL_350C0974E Org_Taestivumearly-release transcript: Traes_4DL_350C0974E.2 (M=6) PTHR11668:SF259 - SERINE/THREONINE-PROTEIN PHOSPHATASE PP1 ISOZYME 2-RELATED (PAC:31968860)

CGGCCCACCACCCAATCTCTCTCCTCCCCCTCCTCGGCCACCTGCTCCTCCCGAAGCGCCGAAACCCTAACTCCACCGATCTCTCTCCACCCCGGGGGAGGCGATGGCGGCGGCGCCGGCGGCGGGAGGGCAGGGAGGCGGCATGGACGCCGCGCTCCTCGACGACATCATCCGCCGTCTGCTCGAGGTGCGGACGGCGCGCCCCGGCAAGCAGGTGCAGCTCTCCGAGTCGGAGATCCGCCAGCTCTGCACCGCCTCCCGCGACATCTTCCTCACCCAGCCCAACCTCCTCGAGCTCGAGGCGCCCATTAAAATCTGCGGTGATATCCATGGTCAGTACAGTGATCTTTTAAGGCTATTTGAGTATGGAGGTTTTCCCCCGGAAGCCAACTATCTATTCTTAGGCGATTATGTTGATCGAGGCAAACAGAGTCTGGAGACTATATGCCTCCTCCTTGCATACAAAATCAAGTACCCTGAGAACTTTTTTCTTCTGAGAGGCAACCATGAGTGTGCTTCAATAAACAGAATATATGGATTTTATGATGAATGCAAGCGTCGCTTCAATGTGCGGCTATGGAAGGTCTTCACCGACTGTTTTAACTGTCTCCCTGTGGCCGCTCTAATTGATGATAAAATATTATGCATGCATGGTGGCCTTTCTCCTGATCTGGGGCACCTAGATGAGATAAAAAACTTGCCCCGTCCTACCGATGTGCCAGATACAGGTCTACTATGCGATCTTCTTTGGTCTGATCCAGGAAAAGATGTCCAAGGGTGGGGCATGAATGATAGGGGCGTTTCATACACATTTGGCCATGACAAAGTTACGGAGTTCCTTCTAAAGCATGATCTTGATCTTATTTGCCGTGCCCACCAGGTTGTTGAGGATGGGTATGAATTCTTTGCTGACAGACAGCTGGTCACCATATTTTCGGCTCCCAACTATTGTGGTGAATTTGATAATGCTGGAGCAATGATGAGTGTTGATGAAACTTTGATGTGTTCATTTCAAATTCTCAAACCTGCTGAGAGAAAAATCAAATTTATGGCGTCAAACAAGATGTGAAATGATGAAGCTCTTTCCTCTTCTAGAGGCTCTGAGGCTCTCATGTGGACACAATCACATCAAGACATCAACGCGCAGGCGCTATGCTTGAAGGCAGCATCGGGCTAGTTCTACTGGAGAAGTCAAATAATATCATTTCTAACAGTTGGTCTGAAAGTTACTCTTCATAGCAAGTGCCAGAATTTTTTCCTTTTCTGTTCCCACCGGGTGTGAAACTATCTTGTAGCTGTAGATATATTGATTATGGAAGGCTGAAGTTCGCAATTGACGCAAGATGATCCCCTCCAGCAAGAAAAAAAAAGTTCTGAATATGAGAGTTTTCTGTTTGGGAGTTCCACTATTTAAAAAGCACCTGTATCACTTACTGAGGTAAGAACAAAAGCCAAGCCTGTGTTTCTGTTGCTGTAAGCAAATATTGGATGAGTTTTATTTTAGTTTTTAGAGAGCGGTAATGGTAACAATTCAAATAGTATGAGCACAAAGAATGCTGATGCTTGGTTGGAACTTGATAGAAAATATTCAGCATGTTCCTGATGTACGCTTCACCTTTCAAGTGCTGGATGGCTTTGAGTTTTTGTTCAATCAGACCAGCATGATGGACCGATGCTTGATTAGCATTAACGTGGAAACAAAA

>Traes_4DL_8B9F13EA5 Org_Taestivumearly-release transcript: Traes_4DL_8B9F13EA5.1 (M=10) PTHR11668:SF280 - SERINE/THREONINE-PROTEIN PHOSPHATASE PP1 ISOZYME 7 (PAC:31806780)

ACTCGCCAGGCCTCCCCTCCCTCTTTCGGGAGCGAGAAAAGAAAAGCAGCTGGGTGCGCGCCCCGCACCTATCAGCCCTGCCTCGTCTGCCGGTGCCAGGGGATCCTGCCTGGCTGTCTGCCCGCGCGCGCGCTGGCTGGGCGGATGGCGGGCTCGTCTTCGGCGTCGGGCGCTGTCGACGTCGACCGGATAATCACCAAGCTGCTCGAGGTGCGCGGCGCGCGCCCTGGCAAGCAGGTGAACCTGCTCGAGTCAGAGATCCGAGCACTCTGCACGACCGCCCGCGAGGTCTTTCTGAAGCAGCCGACGCTGCTCGAGCTGGAGGCGCCAATCAAGATCTGCGGCGACCTGCACGGGCAGTACTTTGACCTGCTGCGCCTCTTTGAGTACGGCGGCTTCCCTCCCGACTCAAACTACCTCTTTCTGGGTGACTACGTGGACCGCGGGAAGCAGTCGCTCGAGACCATCTGCCTGCTGCTCGCGTACAAGATCAAGTACCCGGAGAACTTCTTCCTGCTGCGCGGCAACCACGAGTGCGCCTCGATCAACCGCATCTACGGCTTCTACGACGAGTGCAAGCGGCGCTTCTCGGTGAAGCTGTGGAAGACCTTCACAGACTGCTTCAACTGCTTGCCCGTCGCCGCCGTCGTGGACGAGAAGATCCTGTGCATGCACGGCGGCCTCTCGCCCGACCTCAAGCAGCTGAGCCAGATCGCGCGCCTCGAGCGGCCGACGGACGTGCCGGACCAGGGCCTGCTCTGCGACCTGCTCTGGTCCGACCCGGACAAGGACGTGACGGGGTGGGGCGAGAACGACCGCGGCGTCTCGTACACGTTTGGCGCCGACGTCGTCTCCGACTTTCTCGACGAGCACAACCTCGACCTCGTCTGCCGCGCGCACCAGGTGGTTGAGGACGGCTACGAGTTCTTTGCAAAGCGCCAGCTGGTGACCGTCTTCTCGGCGCCAAACTATTGCGGCGAGTTTGACAATGCGGGCGCGATGATGTCGGTGGACGACACTCTGATGTGCTCCTTCCAGATCCTCAAACCCGCCGAGAAGCGGCCGAAGGGGCAGGCGGCCGCCGCCTCGAGCGGCG

>Traes_5BL_86F86B4A9 Org_Taestivumearly-release transcript: Traes_5BL_86F86B4A9.2 (M=10) PTHR11668:SF280 - SERINE/THREONINE-PROTEIN PHOSPHATASE PP1 ISOZYME 7 (PAC:31794343)

CATACCAGCCAGCAGAAAAGGTGCTAGATATATTGTTCGATCTCTCCTCGCCCGGCCGGCCGACCTTTGGTTGGTTGATGCAAAGTTGCCTAGCTACGCTTGCAGATTAACTATCAAGCCCCATGGTCGTTGTCGTCTACTTATAAGCATCCATTGCTGGACCGTCAGACCTTCAAGTAGCTAGGAGCTCCAGTTGGTTTGCTTGCTTTGATTGGCGATGAACGGTGCGGCGCTAGACAACGTGATCCGGCGGCTGCTGGAGGTGCGGCGGGGGCGGCCGGGGAAGCAGCAAGTGCAGCAGGTGCAGCTGGGCGAGGGGGAGATCCGGCAGCTCTGCGCCGCCGCCAAGGACGTCTTCATGCGCCAGCCCAACCTGCTCCAGCTCGACGCCCCCATCAAGATCGCCGGTGACATCCACGGTCAGTACACGGACCTGCTGCGGCTGTTTGAGCTGGGCGGCTTCCCGCCGCAGCACAAGTACCTGTTCCTGGGCGACTACGTGGACCGCGGCAAGCAGAGCATCGAGACCATCTGCCTGCTGCTCGCCTACAAGCTCCGGTACCCGGAGCACTTCTTCCTCCTCCGCGGCAACCACGAGTGCGCCTCCGTCAACCGCGTCTACGGCTTCTACGACGAGTGCAAGCGCCGCTACTCCGTCCGTCTCTGGCGCCACTTCTCCGACTGCTTCAACTGCATGCCCGTCGCCGCCCTCGTCGAGTCGCGCATCCTCTGCATGCACGGCGGCCTCTCCCCGGACCTCCGCCACGTCCGCGACATCGCCGGCCTCTCCAGGCCCGTCGACGTCCCCGACACCGGCCTCCTCTGCGACCTCCTCTGGTCCGACCCCGGCGGCGCCGCCGGGTGGGGGCCCAACGAGAGGGGCGTGTCGTACACGTTCGGGGCGGACGTGGTGGCGGCGTTCATGGAGAGGCACGACCTGGACCTCGTGTGCCGGGCGCACCAGGTGGTGGAGGACGGCTACGAGTTCTTCGCCGGGCGGCGGATGGTCACCGTCTTCTCCGCGCCCAACTACTGCGGCGAGTTCGACAACGCCGGCGCCCTCATGTGCGTCGACGACGACCTCACCTGCTCCTTCCAGATACTCAAGCCCGTGGACAACAGGCAGCGGCGATTCGCCTTCGGCATGGGATCATCCGCAACTACTACTAGCAGGGGGATCCGATCACCGTGGTGCTAATTAATATGCATCCTTGGTCAAAACGGATGCATGCAATAAACCCATCCATCTTTGATTTCATTACCAATTTTGTATACGAGTTTTCTTGCATGCATGGTGTAACTCGCATAAACAACAGGAAAAGCAACGCTTGTACTATTTTCTTCGGTAATGATAACTCCCAAACGTTCGGGAGTTAAGCATGGCAACCCGAACGGGTTTAGATGGTAAGTTTAGTTTACGTGAGATTAGGGAAACATGGCAATTTTCTGACAAAAAAAGAAAAAGGACGAAGTTGCCATCTTCTATCAACTAAAGTTGCCATCCCCTGTTAACTAAAGTTGCCATATAAAAATGTTCGG

>Traes_5DL_F7F10739D Org_Taestivumearly-release transcript: Traes_5DL_F7F10739D.1 (M=10) PTHR11668:SF280 - SERINE/THREONINE-PROTEIN PHOSPHATASE PP1 ISOZYME 7 (PAC:31898036)

GTGCCGGCCGACCTCCTAAAAGCGCTTTCTTTCCACCTCTTATTCTCGGTCCTTCCCTGTCCTCTCATCTTCCTCCACCCTACTTTTGCTGCTCACACATGTTTTCTTCATTTCTTCAAGAATCTTGCGTTTGTGGTGGTGGTGAGGAGAATCTTTGGCTGGCCTGCTTTGTGTGGTCGCCGCCACTCTGTTAATATTCCTTGAACTAGCTAGCTTGCAACATACTCAGAGCAGAGCAGAGCATAGGGTGCTCCATAGCCCATACCAGCCAGCAGAAAAGATTAATTATCAAGCCTCATGGTCGTCGTCGTCGTCTACTTATAAACATCCATTGCTGGACCGCCAGACCTTCAAGTAGGAGCTCCAGTTGGTTTGCTTGATTGACGATGAACGGTGCGGCGCTAGACAACGTGATCCGGCGGCTGCTGGAGGTGCGGCGGGGGCGGCCGGGGAAGCAGCAAGTGCAGCAGGTGCAGCTGGGCGAGGGGGAGATCCGGCAGCTCTGCGGCGCCGCCAAGGACGTCTTCATGCGCCAGCCCAACCTGCTCCAGCTCGACGCCCCCATCAAGATCGCCGGTGACATCCACGGGCAGTACACGGACCTTATCCGACTGTTTGAGCTGGGCGGCTTCCCGCCGCAGCACAAGTACCTGTTCCTGGGCGACTACGTGGACCGCGGCAAGCAGAGCATCGAGACCATCTGCCTGCTGCTCGCCTACAAGCTTCGGTACCCGGAGCACTTCTTCCTCCTCCGCGGCAACC

>Traes_6AL_CCB16DE7E Org_Taestivumearly-release transcript: Traes_6AL_CCB16DE7E.2 (M=10) PTHR11668:SF280 - SERINE/THREONINE-PROTEIN PHOSPHATASE PP1 ISOZYME 7 (PAC:31906008)

CCCAGCTCAATAAATACCTCCACCAGCACCAGCCATTTTGCTGCAAGCACCAGCACACTTCACAGGCCCTCATCATCTTCCATCCTTCCAAAAACATCACTTGCATAGTTGCATGCAACCCAACAAAAAATCTGACTAGGAAATGTCAAGTGACAAGGACATGGGGGGCCAACACACTTTGCAAAAACAACCCCAGCACTGTCCTTGTGATCTCCAACCAGGGGCCCAGGCACAATAACCAAGCATCCACAAGGGCCTGGTTGCAAAAGTTGCATGCTTTATTATTCCACAAAGTCCAATACTAGTCCTTGGGGAATTGGCACAAGGAGAGAGAAAGTGACAGAGAGAGGTAGAGAGAGAGATAGGGGGAGGGTGGGAGAGGAGACCCTGACCAATGCCAAGAACAAGAAGAGATCAGTGAGAAGGCCAGGCGAAAATCCCCACCGGGCGAGAAGAGACGAGCTCCGGCCTCCTCCGAGAACAAGAACAAGAAGGAATCCCGGAGCACGAGGGAAGAATAAAGCGGGCGGAGGAGACCGGAAAGGCCGAGGAGAAAAAAGCTCTTGATTGATGGAGAAGGGGATGGATCCGGCGCTGCTGGACAACATCATCGCCAGGCTGCTGGAGGTGAAGAGCCTCAAGCCCGGGAAGAACGCGCAGCTGTCCGAGTCGGAGATCAAGCAGCTCTGCGCCGCCTCCAAGGAGATCTTCCTCGCGCAGCCCAACCTCCTGGAGCTCGAGGCCCCCATCAAAATCTGCGGTGATGTCCATGGCCAATATTCTGATCTCCTGAGGCTCTTTGAATATGGTGGATATCCTCCTCAGTCCAACTATCTTTTCTTGGGCGATTATGTGGACCGGGGAAAGCAGAGCCTTGAGACAATATGCCTTCTTTTGGCATATAAGGTCAAGTATCCTGAGAACTTCTTTCTTCTAAGGGGCAACCATGAATGTGCATCAGTAAACCGCATCTATGGGTTCTATGATGAGTGCAAGCGCAGATTCAGTGTAAAACTCTGGAAAACATTTACAGACTGTTTTAACTGCTTACCGGTATCAGCGTTGATAGATGAAAAGATTCTATGTATGCATGGAGGTCTTTCCCCAGAGTTGAACAAGCTGGATCAAATACTCAACCTCAATCGCCCCACAGATGTGCCTGATACTGGGTTACTTTGTGATCTTCTTTGGTCCGATCCTTCGAATGAAGCGCAAGGGTGGGCTATGAATGATCGAGGTGTCTCATATACATTTGGACCTGATAAAGTGGCTGAATTTCTTGAGAAGCATGATTTAGACCTCATCTGCCGAGCCCATCAGGTTGTTGAGGATGGCTACGAGTTCTTTGCTGATCGTCAGCTAGTAACAATATTTTCGGCACCTAATTACTGTGGAGAATTCGATAATGCTGGTGCCATGATGAGTGTAGACGAGACGCTGATGTGCTCCTTCCAAATACTCAAACCCGCAAGGAAAATGTTGCCTGGTTCAACTAATAACAAGTCTGGATTCAAGTCAATGAGAGGATGGTGACGGTGGCACGGCGATTGGTGAGCAAGCTGTGGTCTCATCTGCAGGCAGGCACGTGCCTACTACGAGCGGCTGCGACTAACCGGCGTTTTCGCCGATGCCTCGAGCCCGGACCGGAACAAGAACGCAGATTGTACAACTCTCTCGTCGATGGACTGTACATCGTTCATGTCGGATTAACACTTGTTGTAATGTATTATTGTTTTTCTACCGTGCGGATCTCTTTATCGTACGAGAGGACGATGTGAATGAAACTGTTGCTCCTCCTTCCCCCCCTCTAAATTCGGATTCAGGCAGGCATATACGCCAATAAAAACTGGTAAGAGTCTTGGGTGCTGGCTGTGGGATCTTGAGAGACAGGGA

>Traes_6AL_DC03CC56C Org_Taestivumearly-release transcript: Traes_6AL_DC03CC56C.1 (M=4) PTHR11668//PTHR11668:SF244 - SERINE/THREONINE PROTEIN PHOSPHATASE (PAC:31812504)

ATTAGTGACAGGCTCACCCCCATTTGCCACTCGTACCAGGGCCCTTATTTGGCAGGGCCTTCGGGAGCCAAGCATCGCCGGGCCATAAAACGAACCAAAGCCATGGACACGGGCGCACGGACGCTCTCCACCTCTCCTCCTCTCGCAGCCCGACCTCCTCCACGGCTCGCGGCAAGGAGAGATCGTCGATGGACGGGCACGCGGTGGACGAGCTGATCCGGCGGCTCCTGGACGGGAAGAAGGGCAAGGCGCCCGGCAAGAAGGTGCAGCTGAGCGAGGCGGAGATCCGGCACCTCTGCGTCACCGCCAAGGGGATCTTCCTCTCCCAGCCCAACCTCCTCGAGCTCGAGGCCCCCATCAACGTCTGCGGCGACATCCACGGGCAGTTCTCGGACCTACTCCGGCTGTTCGACTACGGCGGGCTGCCGCCGACGGCCAATTACCTGTTCCTGGGCGACTACGTGGACCGGGGCAAGCAGAGCATCGAGACCATCTGCCTGCTGCTGGCGTACAAGATCAAGTTCCCGGACAACTTCTTCCTGCTGCGGGGCAACCACGAGTGCGCCTCCATCAACCGCATCTACGGCTTCTACGACGAGTGCAAGCGCCGCTTCAGCGTCCGCCTCTGGAAGCTCTTCACCGACTGCTTCAACTGCCTCCCCGTCGCGGCGCTCATCGACGAGAAGATCCTCTGCATGCACGGCGGTCTCTCCCCGGACCTCGACAGCCTCGACCGCATCGCCGAGATCCAGCGCCCCGTCGACGTGCCCGACCAGGGCCTCCTCTGCGACCTCCTCTGGTCCGACCCCGACCGCGAGAGCCCCGGCTGGGGCGAGAACGACCGCGGCGTCTCCTTCACCTTCGGCGCCGACAAGGTCGCCGAGTTCCTCAACAAGCACGACCTCGACCTCATCTGCCGCGCCCACCAGGTCGTGGAGGACGGGTACGAGTTCTTCGCGGACCGGCAGCTGGTCACCATCTTCTCCGCGCCCAACTACTGCGGGGAGTTCAACAACGCCGGCGCGCTGATGAACGTCGACGCCAGCCTGCTCTGCTCCTTCCAGATCCTCAAGCCCTTGAGAGCCAAAGCGCAGGCGGACTAACAGAGTTTTTGTTACTATATTGCTATTAGAGGCTCGGTTAGAATCATGGAGATCTCATAGATTTGCATGCACGTATATATTTCTCCTTGCAGATCCATGGTTCTTTCGAATCGTTTGCTTGTAATTCATTATATATGTGTATGTATATAGTCATATAGAGGCATTCACGTCCACTAGCAAAACGTCATCTAAAAG

>Traes_6BL_93357D848 Org_Taestivumearly-release transcript: Traes_6BL_93357D848.1 (M=10) PTHR11668:SF280 - SERINE/THREONINE-PROTEIN PHOSPHATASE PP1 ISOZYME 7 (PAC:31999898)

ATGGATCCGGCGCTGCTGGACAACATCATCGCCAGGCTGCTGGAGGTGAAGAGCCTCAAGCCCGGGAAGAACGCGCAGCTGTCCGAGTCGGAGATCAAGCAGCTCTGCGCCGCCTCCAAGGAGATCTTCCTCGCGCAGCCCAACCTCCTGGAGCTCGAGGCCCCCATCAAAATCTGCGGTGATGTCCATGGCCAATATTCTGATCTCCTGAGGCTCTTTGAATATGGTGGATATCCTCCTCAGTCCAACTATCTTTTCTTGGGCGATTACGTGGACCGAGGAAAGCAGAGCCTTGAGACAATATGCCTTCTTTTGGCTTATAAGGTCAAGTACCCTGAGAACTTCTTTCTTCTAAGGGGAAACCATGAGTGTGCATCAGTAAACCGCATCTATGGATTCTATGATGAGTGCAAGCGCAGATTCAGTGTGAAACTCTGGAAAACATTTACAGACTGTTTTAACTGCTTACCGGTATCAGCATTGATAGATGAAAAGATTCTATGCATGCATGGAGGTCTTTCCCCAGAGTTGAACAAGCTGGATCAAATACTCAACCTCAATCGCCCCACGGATGTGCCTGATACTGGGTTACTTTGTGATCTTCTTTGGTCCGATCCTTCCAATGAAGCGCAAGGGTGGGCTATGAATGATCGAGGTGTCTCTTATACATTTGGACCTGATAAAGTGGCTGAATTTCTTGAGAAGCATGATTTAGACCTCATCTGCCGAGCCCATCAGGTTGTTGAGGATGGCTACGAGTTCTTTGCTGATCGTCAGCTAGTAACAATATTTTCGGCGCCTAATTACTGTGGAGAATTCGATAATGCTGGTGCCATGATGAGTGTAGACGAGACGCTGATGTGCTCCTTCCAAATACTCAAACCTGCAAGGAAAATGTTGCCTGGTTCAACTAATAACAAGTCTGGCTTCAAGGTATGCATAACTAACATGGGAACAATTGGTATTTTGTCATATACATATATATGTCTCCGTGCTTACCTGCCGTTTCCCTTAAAAATAGCCCCATGCTATATTTACATGCTATTCTGTACAAATATATCATTTGGTTGTCTGTTAACCTGCAAAAGATCTGA

>Traes_6DL_82B22A082 Org_Taestivumearly-release transcript: Traes_6DL_82B22A082.2 (M=10) PTHR11668:SF280 - SERINE/THREONINE-PROTEIN PHOSPHATASE PP1 ISOZYME 7 (PAC:31913764)

ATGGATCCGGCGCTGCTGGACAACATCATCGCCAGGCTGCTGGAGGTGAAGAGCCTCAAGCCCGGGAAGAACGCGCAGCTGTCCGAGTCGGAGATCAAGCAGCTCTGCGCCGCCTCCAAGGAGATCTTCCTCGCGCAGCCCAACCTCCTGGAGCTCGAGGCCCCCATCAAAATCTGCGGTGATGTCCATGGCCAATATTCTGATCTCCTGAGGCTCTTTGAATATGGTGGATATCCTCCTCAGTCGAACTATCTTTTCTTGGGCGATTACGTGGACCGAGGAAAGCAGAGCCTTGAGACAATATGCCTTCTTTTGGCTTATAAGGTCAAGTACCCTGAGAACTTCTTTCTTCTAAGGGGAAACCATGAGTGTGCATCAGTAAACCGCATCTATGGATTCTATGATGAGTGCAAGCGCAGATTCAGTGTGAAACTCTGGAAAACATTTACAGACTGTTTTAACTGCTTACCGGTATCAGCGTTGATAGATGAAAAGATTTTATGTATGCATGGAGGTCTTTCCCCAGAGTTGAACAAGCTGGATCAAATACTCAACCTCAATCGCCCCACGGATGTGCCTGATACTGGGTTACTTTGTGATCTTCTTTGGTCCGATCCTTCGAATGAAGCACAAGGGTGGGCTATGAATGATCGAGGTGTCTCATATACATTTGGACCTGATAAAGTGGCTGAATTTCTTGAGAAGCATGATTTAGACCTCATCTGCCGAGCCCATCAGGTCGTTGAGGATGGCTACGAGTTCTTTGCTGATCGTCAGCTAGTAACAATATTTTCGGCACCTAATTACTGTGGAGAATTCGATAATGCTGGTGCCATGATGAGTGTAGACGAGACGCTGATGTGCTCCTTCCAAATACTCAAACCTGCAAGGAAAATGTTGCCTGGTTCAACTAATAACAAGTCTGGCTTCAAGAAATATATCATTTGGTTGTCTGTTAACCTGCAAAAGATCTGA

>TaPP1g1 TRIAE_CS42_3B_TGACv1_221851_AA0751720.1 A0A1D5VWA1-1

ACGACGAGCCAAAAACGGTGTTCCCGGACGAGGCGTGCGACGATCTCGGCGGCGAGTTCTGCGAGGCGCCCTACCAAACCAGCAAGTAACTAACTCAAGCAGCTACTACGAACTACTATTAGTGTAAAAAACGCTCTTATTCTCAGCAGAGGTCGATCGATGTATATCATGTGAATAAGCAAAAACATGCTGCTACGCTGCTGCCTGTACTGTATTATTGCACATAAACGAAATGCAGTCATCAATTCGTTCGTCTTAAATTCTTAATCCTTCTGTTCCAGTGAACTTAAATCAACGGAAAAATAATTTACAACTAAAAAACGTAATTTCATTTATAGAAACCCGCCCGTTAAAAATTAAAGGTGAGATAGTTGCGGCGGTTTTCAATAAAAAAAAGATAAAAAAATTCCTTAGAAAAAAAAAGAGATAATAATCCCGATCAATCGAGTCCGCTTCCGATCCCAACTACGATCGGCCCGTCCATCCTATTCTACCTACATATACGCTGGCGATCGATCGATCTATCGATCCTGCCCAGCAAATCAAGCAAGCAAGAGTGCAAAACCTACGCGATTCCGATCGATCCAGCAGTAACCAAACTGCTAGAACGCGATCGATGGCGGCCGACTTGGACCTGGACGACGTGATCCAGCGCCTGCTGGACGCGGAGGCGCCCCTCAGCTCGCCGTCCGCCGCCCCGCCGCTGAAAGCCGAGGAGATCCGGCACCTCTGCGCGGCCGCCAAGGAGCTCCTCCTCAAGCAGCCGACGCTGCTCCAGCTCTCCGCCCCCGTCAACATCTGCGGCGACATCCACGGCCAGTACCCCGACCTCCTCCGCCTCTTCCGCGAGATCGGGCCGCCCTCCGCCGCCAACCGCTACCTCTTCCTCGGCGACTACGTCGACCGGGGCACGCAGAGCATCGAGACCATCTGCCTCCTCCTCGCCTACAAGCTCAAGTACCCGGACGCCTTCTTCCTCCTCCGCGGCAACCACGAGTGCGCCGCCGTCAACAAGCAGTACGGCTTCTACTCCGAGTGCGCCTCCCGCGGCCGCCGCATAGTCAGGCTCTGGGAGGAACTCAACGCCGTATTCGCCTGCCTCCCGCTGGCGGCGCTCGTCGGCTGCGAGGGTAGTAACAAGAACAAGAAGAAGAAGATCCTGTGCGTGCACGGCGGGCTCTCGCCGGAGCTCGAGAGCCCGGACCAGATCCGCCAGATCAAGCGCCCGCTGGCCGACGTCCCCGAGCACGGGCTCGTGTGCGACCTGCTGTGGTCGGACCCCGCCGCGGACGGCGACGATTGGGGGTGGGGGGACCCGCGCAGGTGCACGTCCTTCACCTTCGGCGCCGACGTGGTGGAGGAGTTCTGCGAGAGGCACGGGCTGGCCATGGTGTGCAGGGCGCACGAGATGAAGGACGGCGGGTACGACCAAGGGTTCGCCGGCGGGAAGCTTGTCACCGTGTTCTCTGCACCCAACTACTGCGGCAAGTGCGGCAACGACGGCGCCGTCATGACCGTCGCCGGTGACCTCGCCTGCTCCTTCCGCGTCTTCCACCCTGAGAATACTGCCACTCCTCCTCCGGCTCCTATTTATCTTTAGTCCAACATGTATATTCATGTCAGTTTCAGAGTGTAATCTCCAGCTCGTTGTGTCTTCGTGTTCAGAGTGAATTTCAGTTTTCGAATGTATTGCAGGATTTTATCCCATTTAGCGAAAGTTTTACTAGATTTACCACATATAAATTTTCGAGAGTGAT

>TaPP1g3 TRIAE_CS42_3B_TGACv1_224143_AA0792930.1 A0A1D5W4G9-1

TCCGTCCTATTCTACCTACAAATACGCTGGCGATCGATCAATCAATCAAGCAATTCCGATCAATCCTGCAGTAAGAAAAAACAAATGATGGCGGCTGACTTGGACCTGGACGACGTGATCCAGCGCCTCCTGGACGCGGAGGCTCCCCTGAGCTTGCCGTCCGCCGCCCCGCCGCTGAAAGGCGAGGAGATCCGGCACCTCTGCGCGGCCGCCAAGGAGCTCCTCCTCAAGCAGCCGACGCTCCTCGAGCTCTCCGCCCCCATCAACATCTGCGGCGACACCCACGGCCAGTACCCCGACCTCCTCCGCCTCTTCCGCTACAAGCTCAAGTACCCGGACGCCTTCTTCCTCCTCCGCGGCAACCACGAGTGCGCCGCCGTCAACAAGCAGTACGGCTTCTACTCCGAGTGCGCCTCCCGCGGCCGCCGCATAGTCAGGCTCTGGGAGGAACTCAACGCCGTCTTCGCCTGCCTCCCGCTGGCGGCGCTCGTCGGCTGCGATAGTAAAAAGAACAAGAAGAAGAAGATCCTGTGCGTGCACGGCGGGCTCTCGCCGGAGCTCGAGAGCCCGGACCAGATCCGCCAGATCAAGCGCCCGCTGGCCGACGTCCCCGAGTCCGGCCTCGTGTGCGACCTGCTGTGGTCGGACCCCGCCGCGGACGGCGACGACTGGGGGTGGGGGGACCCGCGCAGGAGCACGTCCTTCACCTTCGGCGCCGACGTGGTGGAGGAGTTCTGCGAGAGGCACGGGCTGGCAATGGTGTGCAGGGCGCACGAGATGAAGGATGCCGGGTACGACCAAGAGTTCGCCGGCGGGAAGCTTGTGACCGTGTTCTCTGCGCCCAACTACTGCGGCAAGTGTGGCAACGACGGCGCCGTCATGACCGTGGCCGGTGACCTCGCCTGCTCTTTCCGCGTCTTCCACCCTGATGCTACTGCTGCCACTCCTCCTCCGGCTCCTATTTATCTTCTTTAGTCCTACATACATAATTCAT

>TaPP1f-D TRIAE_CS42_5DL_TGACv1_433240_AA1406660.1 TRIAE_CS42_5DL_TGACv1_433240_AA1406660.1.exon1 exon:protein_coding

TTTTAGTATAGACGGTCACAGTAAGAGAACCACCATATGCGTGCAGGCCGGCACCACAAGGATATATTCGCAACAGCAACAAGAGCTGCTCACTTTCTCCCCGCTTTAAATTTGTGCCGGCCGACCTCCTAAAAGCGCTTTCTTTCCACCTCTTATTCTCGGTCCTTCCCTGTCCTCTCATCTTCCTCCACCCTACTTTTGCTGCTCACACATGTTTTCTTCATTTCTTCAAGAATCTTGCGTTTGTGGTGGTGGTGAGGAGAATCTTTGGCTGGCCTGCTTTGTGTGGTCGCCGCCACTCTGTTAATATTCCTTGAACTAGCTAGCTTGCAACATACTCAGAGCAGAGCAGAGCATAGGGTGCTCCATAGCCCATACCAGCCAGCAGAAAAGGTAATAGATATATATTGTTCGATCTCTCTCTCCGGCCGGCCGACCTTTGGTTGGTTGATGCAAAGTTGCCTAGCTACGCTTGCAGATTAATTATCAAGCCTCATGGTCGTCGTCGTCGTCTACTTATAAACATCCATTGCTGGACCGCCAGACCTTCAAGTAGGAGCTCCAGTTGGTTTGCTTGATTGACGATGAACGGTGCGGCGCTAGACAACGTGATCCGGCGGCTGCTGGAGGTGCGGCGGGGGCGGCCGGGGAAGCAGCAAGTGCAGCAGGTGCAGCTGGGCGAGGGGGAGATCCGGCAGCTCTGCGGCGCCGCCAAGGACGTCTTCATGCGCCAGCCCAACCTGCTCCAGCTCGACGCCCCCATCAAGATCGCCGGTGACATCCACGGGCAGTACACGGACCTTATCCGACTGTTTGAGCTGGGCGGCTTCCCGCCGCAGCACAAGTACCTGTTCCTGGGCGACTACGTGGACCGCGGCAAGCAGAGCATCGAGACCATCTGCCTGCTGCTCGCCTACAAGCTTCGGTACCCGGAGCACTTCTTCCTCCTCCGCGGCAACCACGAGTGCGCCTCCGTCAACCGCGTCTACGGCTTCTACGACGAGTGCAAGCGCCGCTACTCGGTCCGCCTCTGGCGCCACTTCTCCGACTGCTTCAACTGCATGCCCGTCGCCGCCCTCATCGAGTCGCGCATCCTCTGCATGCACGGCGGCCTCTCCCCGGACCTCCGCCACATCCGCGACATCGCTGGCCTCCCCAGGCCCGTCGATGTGCCGGACACCGGCCTCCTCTGTGACCTCCTCTGGTCCGACCCCGGCGGCGCCGCGGGGTGGGGGCCCAATGAGAGGGGCGTGTCGTACACGTTCGGGGCGGACGTGGTGGCGGCGTTCATGGAGAGGCATGACTTGGACCTCGTGTGCCGGGCGCACCAGGTGGTGGAGGACGGCTACGAGTTCTTCGCCGGGCGGAGGATGGTCACGGTCTTCTCGGCCCCCAACTACTGCGGCGAGTTTGATAACGCCGGCGCCCTCATGTGCGTCGACGACGACCTCACCTGCTCGTTTCAGATACTCAAGCCCGCGGACAACAGGCAGCGGCGTTTCGCCTTCGGCATGGGATCATCGGCAACTACTAGTAGGGGGGTCCGATCCCCGTGGTGGTAATTAACACACTTTGGTCAAGACGGATGCAATAAACCCATCTTTGATTTCCTTG

TCAATTTTGTATACTATAAAGTTTCTTGCGTTCATGCATGGTCTAACTCAGATAAACAACAGCAAAAGCAAGCCTTGTAATTTCTTTGGAGGCATCCGGACACTCTTTTCGTAGGGTTAAATAATGTTGTAGTACAAGTTAGTTTTTATATGTGACAAAGGACACACACTTTGCCCACCGTGGGTTTCTTGCCCCTACGCCTATACATTTGCCTTTTAACACACTTTGGTCAAGACGGATGCAATAAACCCATCTTTGATTTCCTTGTCAATTTTGTATACTATAAAGTTTCTTGCGTTCATGCATGGTCTAACTCAGATAAACAACAGCAAAAGCAAGCCTTGTAATTTCTTTGGAGGCATCCGGACACTCTTTTCGTAGGGTTAAATAATGTTGTAGTACAAGTTAGTTTTTATATGTGACAAAGGACACACACTTTGCCCACCGTGGGTTTCTTGCCCCTACGCCTATACATTTGCCTT

>TaPP1g2 TRIAE_CS42_3B_TGACv1_221851_AA0751750.1 A0A1D5VWA3-1

CGTACATCCTATTCTACTTACAAAGATACTCTGGCGATCGATCAATCAATCAAGCAATTCCGATCAATCCTGCAGTAAGAAAAAACTAGTGAGCGATTGATGGCGGCTGACTTGGACCTGGACGACGTGATCCAGCGCCTCCTGGACGCGGAGGCGCCCCTCATCAGCTCGCCGTCCACCGCCCCGCCGCTGAAAGGCGAGGAGATCCGGCACCTCTGCGCGGCCGCCAAGGAGCTGCTCCTCAAGCAGCCGACGCTGCTCGAGCTCTCCGCCCCCATCAACATCTGCGGCGACATCCACGGCCAGTACGCCGACCTCCTCCGCCTCTTCCGCGAGACCGGGCCGCCCTCCGCCGCCAATCGCTACCTCTTCCTCGGCGACTACGTCGACCGGGGCACGCAGAGCATCGAGACCATCTGCCTCCTCCTCGCCTACAAGGTCAAGTACCCGGACGCCTACTTCCTCCTCCGCGGCAACCACGAGTGCGCCGCCGTCAACAAGCAGTACGGCTTCTACTCCGAGTGCGCCTCCCGCGCCCGCCGCATAGTCAGGCTCTGGGAGGAACTCAACGCCGTATTCGCCTGCCTCCCGCTGGCGGCGCTCGTCGGCTGCGATAGTAAAAAGAACAACAAGAAGAAGATCCTGTGCGTGCATGGAGGGCTCTCGCCGGAGCTCGAGAGCCCGGACCAGATCTGCCAGATCAAGCGCCCGCTGGCCGACGTCCCCGAGTCCGGCCTCGTCTGCGACCTGCTGTGGTCGGACCCCGCCGCGGACGGCGACGATTGGGGGTGGGGGGACCCGCGCAGGAGCACGTCCTTCACCTTCGGCGCCGACGTGGTGGAGGAGTTCTGCGAGAGGCACGGGCTGGCCATGGTGTGCAGGGCGCACGAGATGAAGGATGCCGGGTACGACCAAGAGTTCGCCGGCGGGAAGCTTGTGACTGTGTTCTCTGCACCCAACTACTGCGGCAAGTGCGGCAACGACGGCGCCGTCATGACCGTCGCCGGTGACCTCGCCTGCTCCTTCCGCGTCTTCCACCCTGAGACTACTGCCACTCCTCCGGCTCCTATCTGTCTTTAGTCCAACATACATATGTTAGTTCCAGACGCCTTGCCATGTAATCCCCAGCTCGTCGCGTCTTGGATTATTGAGACTGCATAATTTCAGTTTTTAACGCCTAATTCAATTTTTAC
