# Supplementary figures and images for: Genome wide identification of wheat and Brachypodium type one protein phosphatases and functional characterization of durum wheat TdPP1a
Source: PLoS One. 2018 Jan 16;13(1):e0191272. doi: 10.1371/journal.pone.0191272 (PMC5770040; doi:10.1371/journal.pone.0191272)

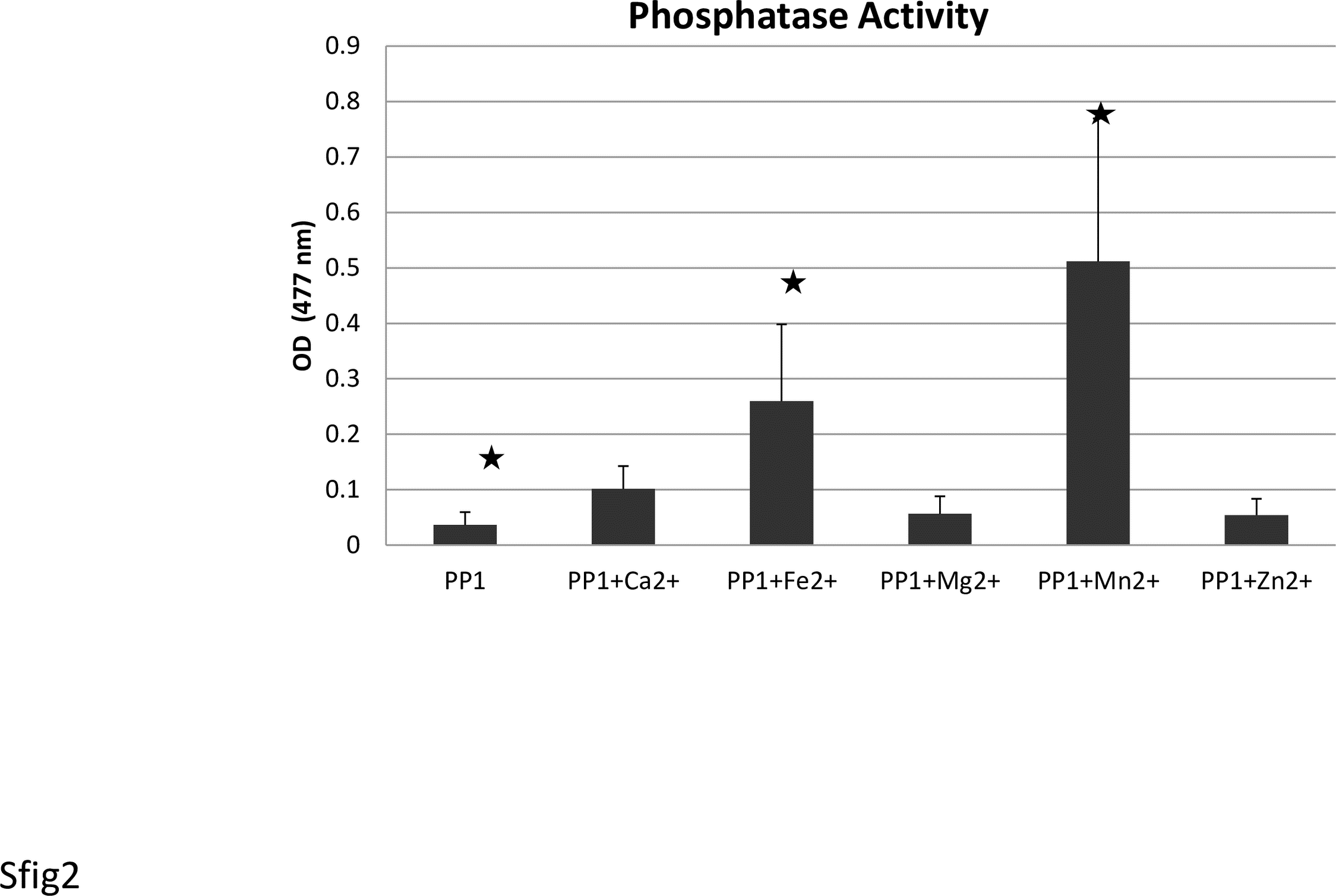

Supplement: S2 Fig — Recombinant His::PP1a phosphatase activity using OMFP as a substrate. Activities registered on 6xHis::TdPP1a protein in absence or in presence of Ca2+, Mg2+, Zn2+, Mn2+ and Fe2+ (1 mM each). Values are means of at least 3 independent experiments (± S.E). Stars represent statistical significance (Student’s T-test p<0.01). (TIF) [file pone.0191272.s002.tif]

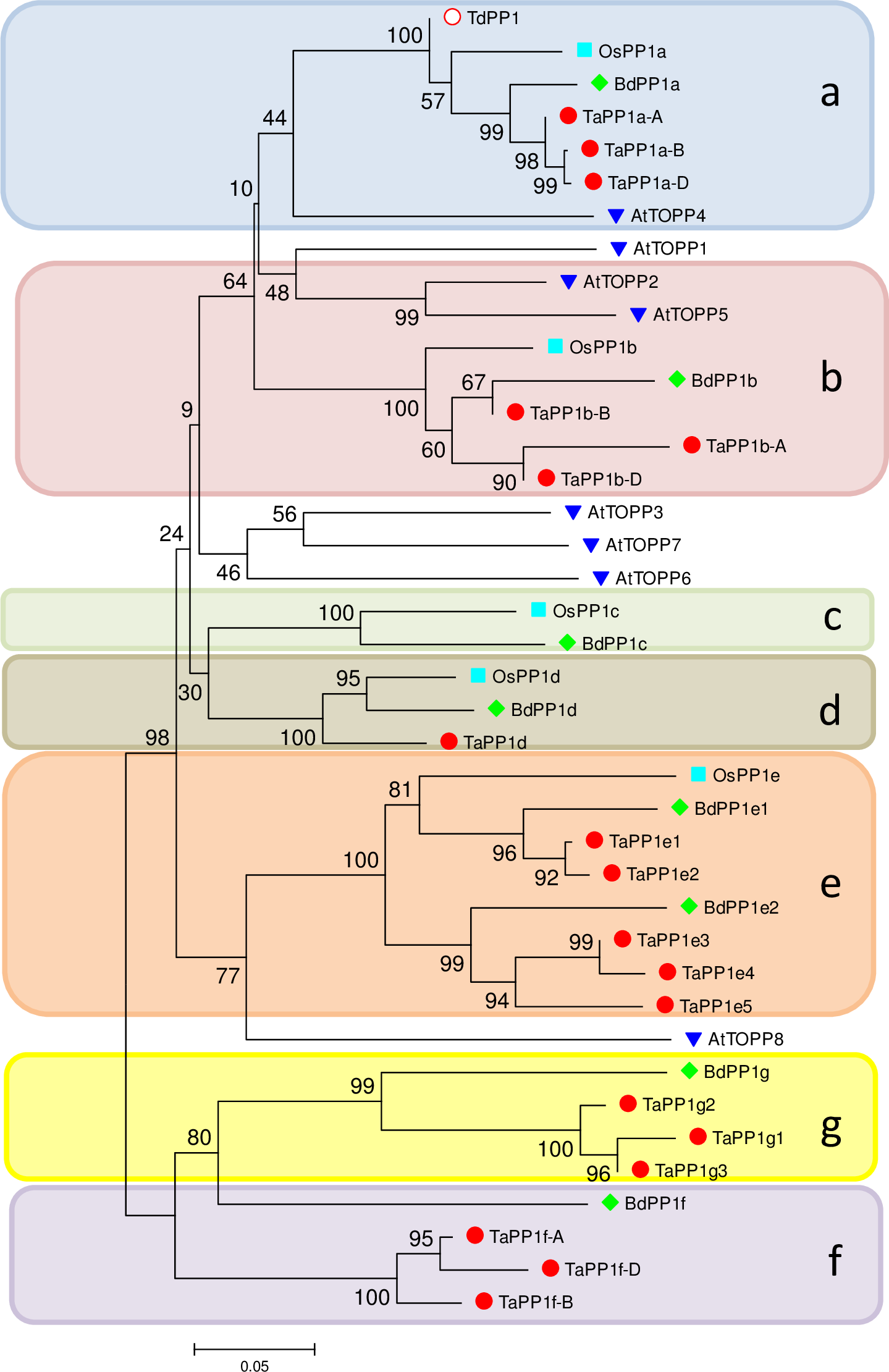

Supplement: S3 Fig — Phylogenetic tree was obtained using MEGA6 with the Neighbor-Joining method based on cDNA sequences from EnsemblPlant, phytozome and TAIR. Wheat sequences are indicated with red dots, rice with light blue squares, Brachypodium PP1 genes with green diamonds and Arabidopsis with dark blue triangle. The percentage of replicate trees (bootstrap test with 1000 replicates) in which each taxa clustered together is indicated on each branch. The tree is drawn to scale and indicates evolutionary distances in number of synonymous to non-synonymous nucleotide substitutions. (TIF) [file pone.0191272.s003.tif]

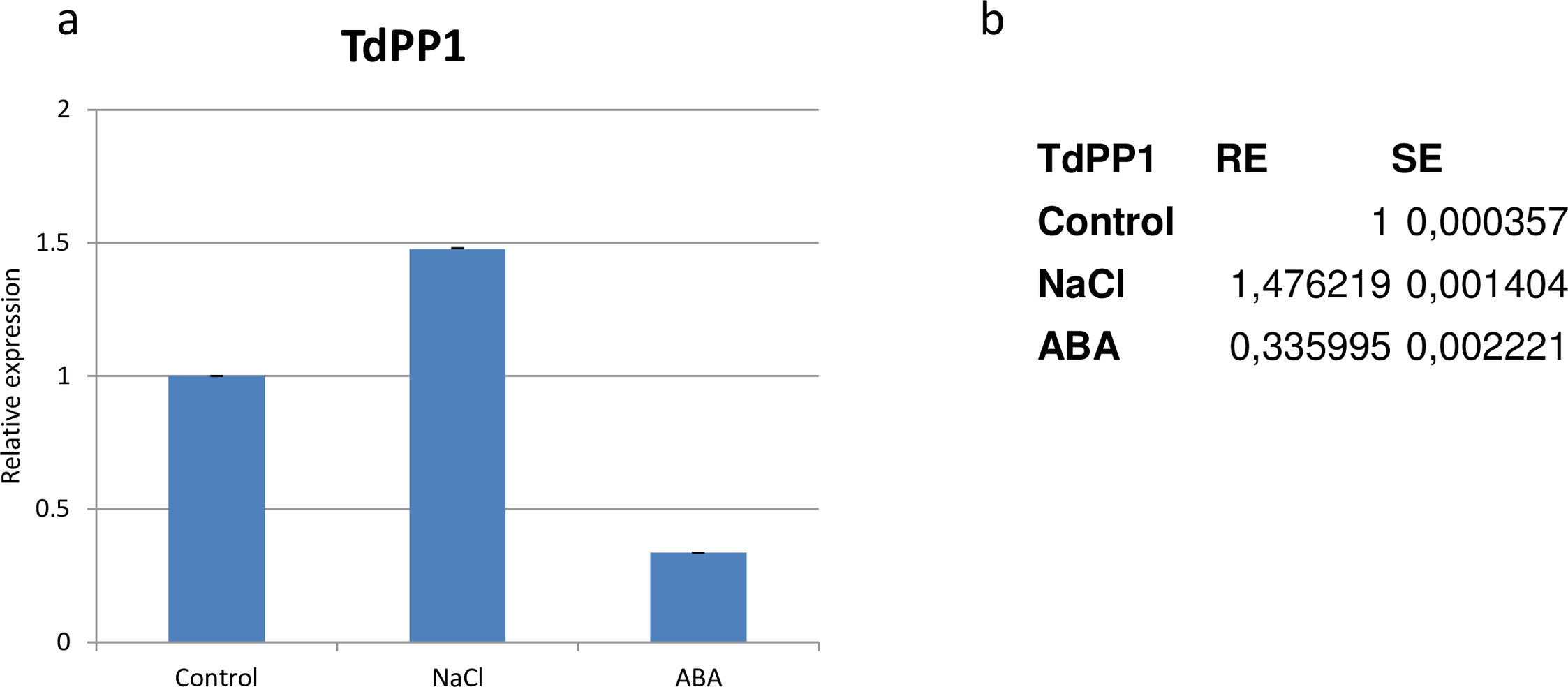

Supplement: S4 Fig — Seven-day-old durum wheat seedlings were treated with NaCl (150 mM), or ABA (100 μM) for 1 hour. (A) Relative expression of TdPP1a was analyzed by quantitative real-time qPCR using wheat actin as control. (B) Raw data of relative expression (RE) with standard error (SE). (TIF) [file pone.0191272.s004.tif]
