# Supplementary material for: Genome wide identification of wheat and Brachypodium type one protein phosphatases and functional characterization of durum wheat TdPP1a
Source: PLoS One. 2018 Jan 16;13(1):e0191272. doi: 10.1371/journal.pone.0191272 (PMC5770040; doi:10.1371/journal.pone.0191272)
Supplement: S1 Table — (DOCX) [file pone.0191272.s005.docx]

Supplemental Table 1: PP1 wheat genes (encoded by A and D genomes) and their orthologs in *Triticum urartu* and *Aegilops tauschii.*

| Wheat ID | *T.urartu* ortholog | % ID | *A.tauschii* ortholog | % ID |
| --- | --- | --- | --- | --- |
| Traes_1AS_4025A300E | [TRIUR3_29448-P1](http://plants.ensembl.org/Triticum_urartu/Transcript/ProteinSummary?db=core;t=TRIUR3_29448-P1;tl=b1qV7OpgrFkqcRkC-18126697-332893440) | 89.8 % | nd | nd |
| Traes_1DS_309F807A1 | nd | nd | [EMT16170](http://plants.ensembl.org/Aegilops_tauschii/Transcript/ProteinSummary?db=core;t=EMT16170;tl=PBkvWFfsRDwkGAcr-18126700-332893551) | 100% |
| Traes_3AS_8B6A13B23 | [TRIUR3_29448-P1](http://plants.ensembl.org/Triticum_urartu/Transcript/ProteinSummary?db=core;t=TRIUR3_29448-P1;tl=1TUL2hWB3VkLe0HW-18126703-332893698) | 99.6 % |  |  |
| Traes_4AS_6D7CDA716 | [TRIUR3_27093-P1](http://plants.ensembl.org/Triticum_urartu/Transcript/ProteinSummary?db=core;t=TRIUR3_27093-P1;tl=UD5bKcVd55aUwVkg-18126707-332893855) | 99.7% |  |  |
| Traes_4DL_350C0974E |  |  | [EMT10824](http://plants.ensembl.org/Aegilops_tauschii/Transcript/ProteinSummary?db=core;t=EMT10824;tl=zg26g9jcHBChi2ZT-18126711-332894022) | 100 % |
| Traes_4DL_8B9F13EA5 |  |  | [EMT10824](http://plants.ensembl.org/Aegilops_tauschii/Transcript/ProteinSummary?db=core;t=EMT10824;tl=tB3F6BSNR4OVP8Ih-18126717-332894133) | 100 % |
| Traes_6DL_82B22A082 |  |  | [EMT09882](http://plants.ensembl.org/Aegilops_tauschii/Transcript/ProteinSummary?db=core;t=EMT09882;tl=7geykjOm5UkXY82G-18126691-332892923) | 100 % |
| Traes_6AL_CCB16DE7E | [TRIUR3_17571-P1](http://plants.ensembl.org/Triticum_urartu/Transcript/ProteinSummary?db=core;t=TRIUR3_17571-P1;tl=1xsSKBt337koQP1o-18126738-332895901) | 100 % |  |  |
| Traes_6DL_82B22A082 |  |  | [EMT09882](http://plants.ensembl.org/Aegilops_tauschii/Transcript/ProteinSummary?db=core;t=EMT09882;tl=MaAwWaE1fa7HYUzF-18126743-332896180) | 100 % |

Accession numbers indicated are from EnsemblPlant. % ID between cDNA sequences was calculated using BLAST.
