# Supplementary material for: Genome wide identification of wheat and Brachypodium type one protein phosphatases and functional characterization of durum wheat TdPP1a
Source: PLoS One. 2018 Jan 16;13(1):e0191272. doi: 10.1371/journal.pone.0191272 (PMC5770040; doi:10.1371/journal.pone.0191272)
Supplement: S2 Table — Accession number, size, molecular weight, pI, instability index and GRAVY. The latter prarameters were predicted with protparam. (DOCX) [file pone.0191272.s006.docx]

Supplementary Table 2: Wheat, Brachypodium and rice PP1 proteins with their accession number, size, molecular weight, pI, instability index and GRAVY

| Gene Name | Gene ID | Protein size (aa) | Uniprot ID | Molecular weight | pI | Instability Index | GRAVY |
| --- | --- | --- | --- | --- | --- | --- | --- |
| TaPP1a-A | Traes_4AS_6D7CDA716 | 321 | W5DXV3 | 36.170 | 5.16 | 41.69 | -0.138 |
| TaPP1a-B | Traes_4BL_3AA55AD10 | 320 | W5E2N6 | 36.123 | 5.16 | 41.39 | -0.137 |
| TaPP1a-D | Traes_4DL_350C0974E | 321 | W5DXV3 | 36.170 | 5.16 | 41.69 | -0.138 |
| TaPP1b-A | Traes_6AL_CCB16DE7E | 320 | W5GAA2 | 36.177 | 5.19 | 39.92 | -0.220 |
| TaPP1b-B | Traes_6BL_93357D848 | 320 | nd | 36.178 | 5.19 | 42.97 | -0.025 |
| TaPP1b-D | Traes_6DL_82B22A082 | 324 | W5GXJ4 | 36.714 | 5.28 | 40.06 | -0.162 |
| TaPP1d | Traes_6AL_DC03CC56C | 304 | W5GAP6 | 34.140 | 5.14 | 36.28 | -0.106 |
| TaPP1e1 | Traes_3AS_8B6A13B23 | 325 | nd | 35.896 | 5.21 | 41.99 | -0.153 |
| TaPP1e2 | Traes_3B_9B97F74FC | 352 | W5D0D0 | 39.548 | 5.30 | 41.37 | -0.144 |
| TaPP1e3 | Traes_1AS_4025A300E | 325 | W4ZVQ0 | 36.545 | 5.13 | 41.95 | -0.135 |
| TaPP1e4 | Traes_1BS_BF71914E7 | 325 | W5AB35 | 36.520 | 5.13 | 42.58 | -0.166 |
| TaPP1e5 | Traes_1DS_309F807A1 | 335 | W5AKP0 | 37.918 | 5.21 | 44.21 | -0.119 |
| TaPP1f-A | Traes_4AL_2EBB63DBA | 326 | W5DMS3 | 36.690 | 7.08 | 50.15 | -0.197 |
| TaPP1f-B | Traes_5BL_86F86B4A9 | 326 | A0A096USJ2 | 36.722 | 7.08 | 49.68 | -0.183 |
| TaPP1f-D | [TRIAE_CS42_5DL_TGACv1_433240_AA1406660.1](http://www.ensemblgenomes.org/id/TRIAE_CS42_5DL_TGACv1_433240_AA1406660.1) | 325 | A0A1D5ZL74 | 36.686 | 7.08 | 51.89 | -0.205 |
| TaPP1g1 | TRIAE_CS42_3B_TGACv1_221851_AA0751720.1 | 327 | A0A1D5VWA1 | 35.751 | 5.42 | 51.76 | -0.107 |
| TaPP1g2 | [TRIAE_CS42_3B_TGACv1_221851_AA0751750.1](http://www.ensemblgenomes.org/id/TRIAE_CS42_3B_TGACv1_221851_AA0751750.1) | 326 | A0A1D5VWA3 | 35.600 | 5.09 | 51.30 | -0.075 |
| TaPP1g3 | [TRIAE_CS42_3B_TGACv1_224143_AA0792930.1](http://www.ensemblgenomes.org/id/TRIAE_CS42_3B_TGACv1_224143_AA0792930.1) | 296 | A0A1D5W4G9 | 32.409 | 5.43 | 49.18 | -0.115 |
| BdPP1a | Bradi1g66970 | 321 | I1H733-1 | 36.203 | 5.17 | 42.67 | -0.156 |
| BdPP1b | Bradi3g55614 | 316 | A0A0Q3IMD5-1 | 35.657 | 5.17 | 36.88 | -0.194 |
| BdPP1c | Bradi1g48410 | 325 | I1H0Q6-1 | 35.586 | 5.39 | 40.28 | -0.212 |
| BdPP1d | Bradi3g37570 | 305 | I1I7M3-1 | 34.424 | 5.09 | 41.33 | -0.177 |
| BdPP1e1 | Bradi2g12650 | 325 | I1HF72-1 | 36.558 | 5.30 | 41.64 | -0.183 |
| BdPP1e2 | Bradi2g35150 | 325 | I1HLN1-1 | 36.676 | 5.29 | 53.92 | -0.278 |
| BdPP1f | Bradi1g00690 | 326 | I1GKJ4-1 | 36.694 | 7.05 | 45.97 | -0.107 |
| BdPP1g | Bradi2g03597 | 342 | I1HC56-1 | 36.944 | 5.41 | 48.18 | +0.008 |
| OsPP1a | Os03g0268000 | 322 | P48489 | 36.166 | 5.03 | 42.55 | -0.147 |
| OsPP1b | Os02g0820000 | 316 | Q6K9Q5 | 35.832 | 5.25 | 41.86 | -0.228 |
| OsPP1c | Os06g0164100 | 322 | Q5VRS7 | 36.185 | 5.48 | 39.94 | -0.192 |
| OsPP1d | Os08g0455600 | 307 | Q6Z0R3 | 34.614 | 5.01 | 36.39 | -0.136 |
| OsPP1e | Os01g0349400 | 325 | Q0JMY6 | 36.488 | 5.39 | 41.39 | -0.207 |
