# Supplementary material for: Genome wide identification of wheat and Brachypodium type one protein phosphatases and functional characterization of durum wheat TdPP1a
Source: PLoS One. 2018 Jan 16;13(1):e0191272. doi: 10.1371/journal.pone.0191272 (PMC5770040; doi:10.1371/journal.pone.0191272)
Supplement: S3 Table — Ka/Ks ratios were calculated using MEGA6.06. (DOCX) [file pone.0191272.s007.docx]

Supplementary Table 3: Evolutionary pairwise distances between wheat, Brachypodium, and rice PP1s by calculation of the Ka/Ks ratios.

| **Genes** | **A genome** | **B genome** | **D genome** | **BdPP1a** | **OsPP1a** |
| --- | --- | --- | --- | --- | --- |
| **TdPP1a** | 0.016 | 0 | 0.030 | 0.032 | 0.057 |
| **BdPP1a** | 0.103 | 0.223 | 0.213 | - | - |
| **BdPP1b** | 0.570 | 0.202 | 0.086 | - | - |
| **BdPP1d** | 0.495 | - | - | - | - |
| **BdPP1e1** | 0.274 | 0.320 | - | - | - |
| **BdPP1e2** | 0.352 | 0.418 | 0.423 | - | - |
| **BdPPf** | 0.746 | 0.756 | 0.806 | - | - |
| **BdPP1g** | - | 0.626-0.582-0.570 | - | - | - |
| **OsPP1a** | 0.169 | 0.269 | 0.263 | 0.283 | - |
| **OsPP1b** | 0.270 | 0.178 | 0.113 | - | - |
| **OsPP1d** | 0.398 | - | - | - | - |
| **OsPP1e** | 0.166 | 0.219 | - | - | - |
