# Supplementary material for: Genome wide identification of wheat and Brachypodium type one protein phosphatases and functional characterization of durum wheat TdPP1a
Source: PLoS One. 2018 Jan 16;13(1):e0191272. doi: 10.1371/journal.pone.0191272 (PMC5770040; doi:10.1371/journal.pone.0191272)
Supplement: S4 Table — (DOCX) [file pone.0191272.s008.docx]

Supplementary Table 4: Most relevant cis-elements found in promoters of representative wheat genes^a^

| *Cis*-elements | Consensus sequence | Reference | Number of elements^b^ |
| --- | --- | --- | --- |
| ABRELATERD1 | ACGTG | [TF_motif_seq_0249](http://plantpan2.itps.ncku.edu.tw/TFBSinfo.php?matrix=TF_motif_seq_0249) | 12-17 |
| MYB1AT | WAACCA | TF_motif_seq_0341 | 1-5 |
| LTRECOREATCOR15 | CCGAC | TF_motif_seq_0258 | 10-23 |
| SURECOREATSULTR11 | GAGAC | TF_motif_seq_0261 | 8-32 |

^a^ Representative wheat gene used for this study are TdPP1a-A, TdPP1b-A, TdPP1d, TdPP1e1, TdPP1e4, TdPP1f-A

^b^ The number of each cis-regulatory element is variable among the representative wheat genes as indicated.
